# Phenotyping of Silique Morphology in Oilseed Rape Using Skeletonization with Hierarchical Segmentation

**DOI:** 10.34133/plantphenomics.0027

**Published:** 2023-03-15

**Authors:** Zhihong Ma, Ruiming Du, Jiayang Xie, Dawei Sun, Hui Fang, Lixi Jiang, Haiyan Cen

**Affiliations:** ^1^ College of Biosystems Engineering and Food Science, Zhejiang University, Hangzhou 310058, P.R. China.; ^2^ Key Laboratory of Spectroscopy Sensing, Ministry of Agriculture and Rural Affairs, Hangzhou 310058, P.R. China.; ^3^ Institute of Crop Science and Zhejiang Key Laboratory of Crop Germplasm, Zhejiang University, Hangzhou 310058, P.R. China.

## Abstract

Silique morphology is an important trait that determines the yield output of oilseed rape (*Brassica napus L*.). Segmenting siliques and quantifying traits are challenging because of the complicated structure of an oilseed rape plant at the reproductive stage. This study aims to develop an accurate method in which a skeletonization algorithm was combined with the hierarchical segmentation (SHS) algorithm to separate siliques from the whole plant using 3-dimensional (3D) point clouds. We combined the L1-median skeleton with the random sample consensus for iteratively extracting skeleton points and optimized the skeleton based on information such as distance, angle, and direction from neighborhood points. Density-based spatial clustering of applications with noise and weighted unidirectional graph were used to achieve hierarchical segmentation of siliques. Using the SHS, we quantified the silique number (SN), silique length (SL), and silique volume (SV) automatically based on the geometric rules. The proposed method was tested with the oilseed rape plants at the mature stage grown in a greenhouse and field. We found that our method showed good performance in silique segmentation and phenotypic extraction with *R*^2^ values of 0.922 and 0.934 for SN and total SL, respectively. Additionally, SN, total SL, and total SV had the statistical significance of correlations with the yield of a plant, with *R* values of 0.935, 0.916, and 0.897, respectively. Overall, the SHS algorithm is accurate, efficient, and robust for the segmentation of siliques and extraction of silique morphological parameters, which is promising for high-throughput silique phenotyping in oilseed rape breeding.

## Introduction

Oilseed rape (*Brassica napus L.*) as an important oil source of food [1,2] is one of the most important oil industrial crops worldwide. The yield and quality of oilseed rape are largely determined by the development of siliques, i.e., the number, length, and volume of siliques at the mature stage, because the silique is one of the important organs of an oilseed rape plant, playing a role not only in its bearing of oilseeds but also in photosynthesis and delivering developmental signals to maturing seeds [3–5]. Therefore, breeding a genotype with a large number of siliques and an ideal silique appearance is a prioritized goal for high-yield breeding. The number of siliques varies significantly among different genotypes and under different cultivation practices; therefore, the accurate quantification of the parameters regarding silique development is critical for yield prediction and breeding high-yielding oilseed rape varieties [6,7].

Currently, the traditional measurement of silique development parameters such as silique length (SL) and silique number (SN) depends largely on manual work, which is invasive, time-consuming, and inaccurate [8]. With the development of computer vision technologies, effective and image-based approaches have emerged to cope with the above problems. To date, methods have been developed to determine silique parameters based on 2-dimensional (2D) or 3-dimensional (3D) data. Of these, 2D images have been the most widely used data thus far to phenotype a plant. Liu et al. [8] cut off the siliques from the plants and placed them on the conveyor belt; then, an RGB camera was used to obtain 2D images from the topside. The silique was detected by the concave point detection segmentation method and measured by the pose estimation method. Wang et al. [9] cut and took photos of stems with and without siliques. The morphological appearances were then marked, and the angle between the silique and main stem was measured using AutoCAD software. However, because they are restricted by illumination problems and the one-sided perspective inherent to 2D imaging [10], methods based on 2D image data are less robust and difficult to use to obtain the complete spatial information of plants. The above methods are limited to obtaining simple morphological parameters and cannot be widely applied. In contrast, 3D data acquired using different 3D sensors, such as time-of-flight cameras and laser scanners [11], can represent a more detailed spatial information of the targets, ensuring more high-throughput plant phenotyping.

Clustering-based algorithms are the most widely used methods for 3D plant phenotyping. Xu et al. [12] used an RGB-D camera to obtain reconstructed point clouds of oilseed rapes. They used morphological operations (dilation and erosion) to segment stems and siliques and applied the clustering algorithm to extract the siliques. Lin et al. [13] obtained oilseed rape point clouds of high quality using a laser scanner. The region growing-based clustering algorithm with normal information was applied to segment siliques and stems. However, clustering-based methods are generally applied to plants with population scales or simple structures [14–16], and the performance is highly influenced by the initial value setting and tedious parameter tuning. Moreover, clustering-based algorithms have a limited understanding of the contextual information of the data, making it less reliable to analyze the topological relation between plant skeletons and plant organs.

The skeleton extraction algorithm has been proposed to tackle the above challenge in 3D plant phenotyping analysis due to its ability to depict the plant skeleton and the details of a plant structure [17]. Many researchers have applied skeleton data to analyze morphological parameters. Wu et al. [18] used Laplacian contraction to extract the skeleton of maize data by multiple-view stereo reconstruction and the nearest neighbor clustering method to separate the leaves and calculate the height, leaf length, and leaf tilt angle. Gaillard et al. [19] obtained the skeleton of sorghum by a thinning algorithm based on voxel data. On the basis of the skeleton model, they compared the classification performance between support vector machine, support vector machine with radial-based function, and multilayer perceptron. Because maize and sorghum have simple structures, these methods ignore the influence of occlusion caused by complex structures with multiple branches on skeleton extraction. For plants with complex structures such as trees, skeleton algorithms have also been widely applied. Chaudhury and Godin [20] used B-spline curve lines with an expectation–maximization algorithm to extract and optimize the skeletons of trees. Preuksakarn et al. [21] extracted the skeleton of apple trees by the space colonization algorithm. Li et al. [22] used the *k*-means method and clustering method to separate the branches and main trunk and consequently calculated the skeleton points by breadth-first search in the undirected graph of *Toona* trees and peach trees. They constructed a skeleton model fitting trees well. However, the current methods for skeleton extraction vary because of the experimental conditions and the complexity of the plant structure, and some of them require prior knowledge and labor-intensive manual optimization. In addition, there is less occlusion between tree branches, which is significantly different from oilseed rape. The current skeleton methods cannot be generalized to analyze the oilseed rape directly. Therefore, developing an accurate method for skeleton extraction and silique segmentation of oilseed rape point clouds is necessary for phenotyping studies.

In this study, we proposed a novel algorithm by skeletonization combined with hierarchical segmentation (SHS) on point clouds of a complete oilseed rape at the mature stage, which has not been reported to our knowledge. The goals were to (a) develop the skeleton model of oilseed rape, (b) achieve accurate segmentation of siliques, and (c) obtain the morphological parameters of siliques for yield prediction. Eventually, we hope to introduce a noninvasive phenotyping method with morphological parameters that could be applied to real production.

## Materials and Methods

### Plant materials

Eight oilseed rape (*Brassica napus* L.) cultivars were selected for the study, with 3 biological replicates in this study. Three cultivars (c1 to c3) were grown in pots in the greenhouse at the Zijingang Campus of Zhejiang University, Zhejiang Province, China (30°18′N, 120°6′E). Five cultivars (c4 to c8) were grown in the field at the Changxing Agricultural Experiment Station of Zhejiang University, Zhejiang Province, China (30°53′N, 119°38′E). Basically, these oilseed rapes were divided into 3 types based on the structure of the branches: the plants of few-branch broom shape (FBBS), which only had few branches, and the canopy of the plant was spread out and looked like a broom; the plants of multibranch broom shape (MBBS), which had more branches than FBBS; and the plants of multibranch cylinder shape (MBCS), which had multiple branches, and the canopy of the plant looked like a cylinder. 3D point clouds of the whole plants at the silique mature stage were acquired using a handheld 3D laser scanner. The SN, SL, and seed weight (SW) were measured. The SN was counted manually, and the SL was measured with a tape measure. The seeds of each silique were naturally dried before weighing using an electronic balance (BSA224S, Sartorius, Germany). The details of the cultivars and measured parameters are summarized in Table 1.

**Table 1.** Summary of varieties and parameters of oilseed rape.

Growth condition	Cultivar name	Origin	Replicate #	Manually measured parameters
Greenhouse	ZD619 (c1)	China	3	SN, SL, and SW
	ZD622 (c2)	China	3	
	ZD630 (c3)	China	3	
Field	Sl512 (c4)	Czechoslovakia	3	SN and SW
	Bnw1.61/83 (c5)	Germany	3	
	8426016 (c6)	China	3	
	CR3168 (c7)	Germany	3	
	Hu135 (c8)	China	3	

SN, SL, and SW represent the silique number, silique length, and seed weight, respectively.

### Point cloud data acquisition

**Fig. 1.**
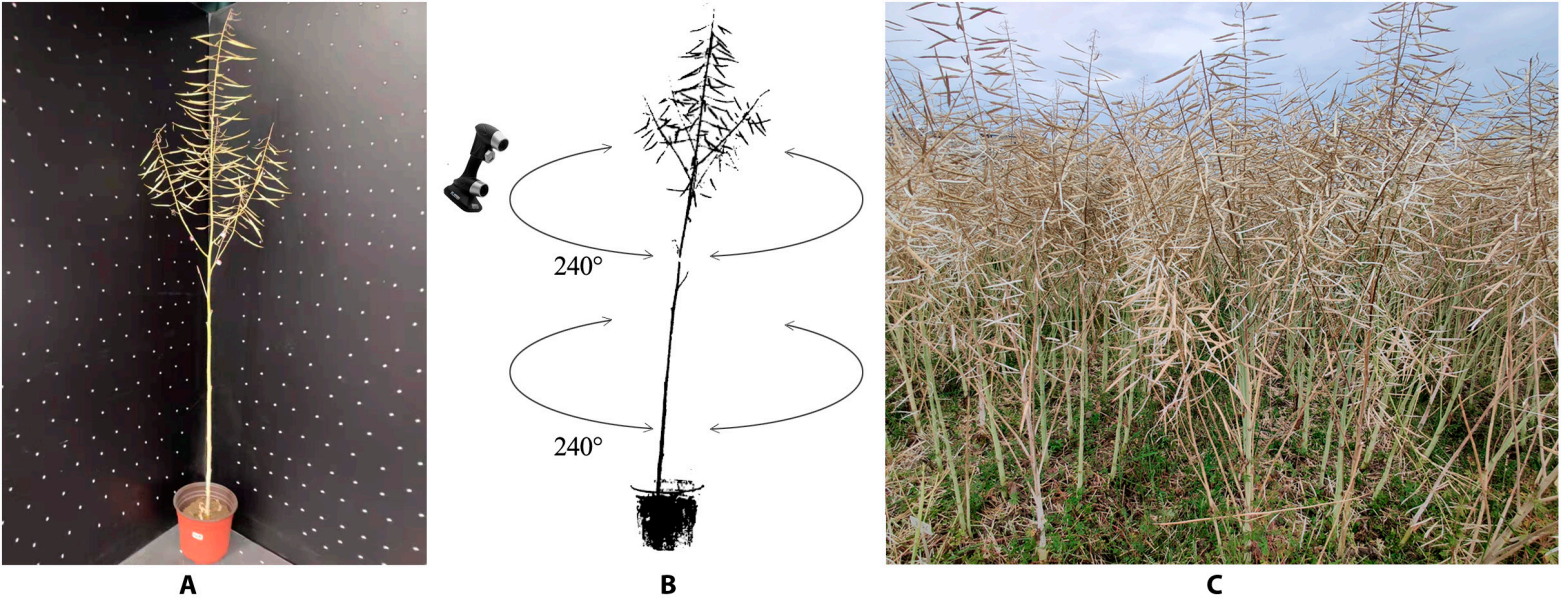
The acquisition of point cloud data. (A) The acquisition environment (plant and 2 blackboards with marker dots). (B) The acquisition process with the laser scanner. (C) The field-grown plants.

A handheld laser scanner (PRINCE775 Inc., SCAN Tech., Hangzhou, China) with 15 red laser beams was used to harvest 3D point cloud data (Fig. 1), and the spatial resolution was 0.05 mm. The plants in pots were scanned in the laboratory, while the field-grown plants were transported to the laboratory for scanning to avoid interference from wind and other plants. The acquisition of raw data involved continuous manual scans from different perspectives. Two blackboards with marker dots provided a coordinate reference for the scanner (Fig. 1B). Because of the limited field of view, the acquired data covered the horizontal range of 240° (Fig. 1C).

### Data processing

**Fig. 2.**
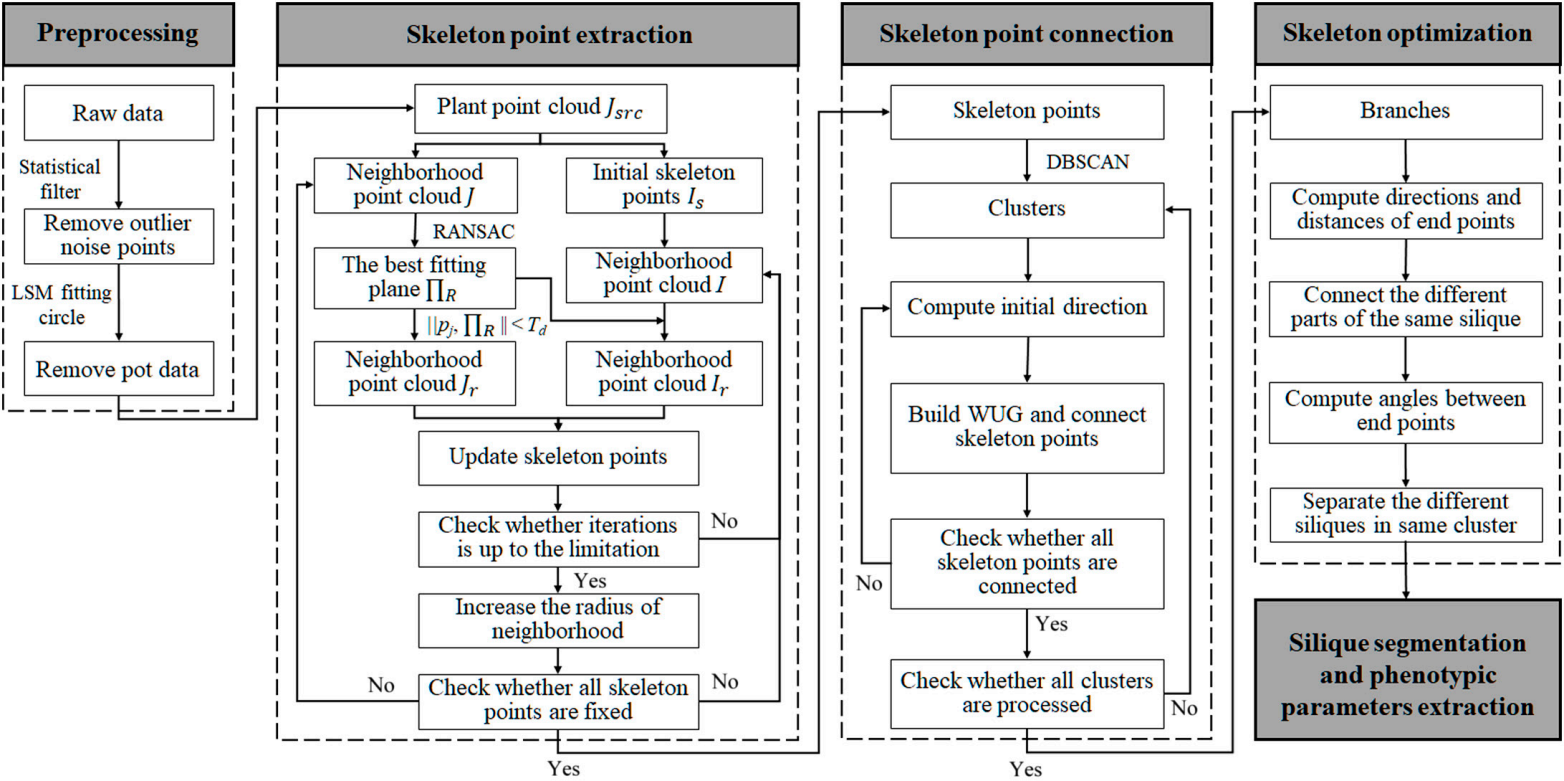
The overall workflow of SHS. *J_src_* is the source point cloud without a pot. *J* is the neighborhood point set in *J_src_*, while *I* is the neighborhood point set in the skeleton point cloud *I_s_*. *J_r_* and *I_r_* are the neighborhood point sets after constraint. ‖*p_j_*, ∏*_R_*‖ is the distance between point *p_j_* and plane ∏*_R_*. *T_d_* is the distance threshold.

**Fig. 3.**
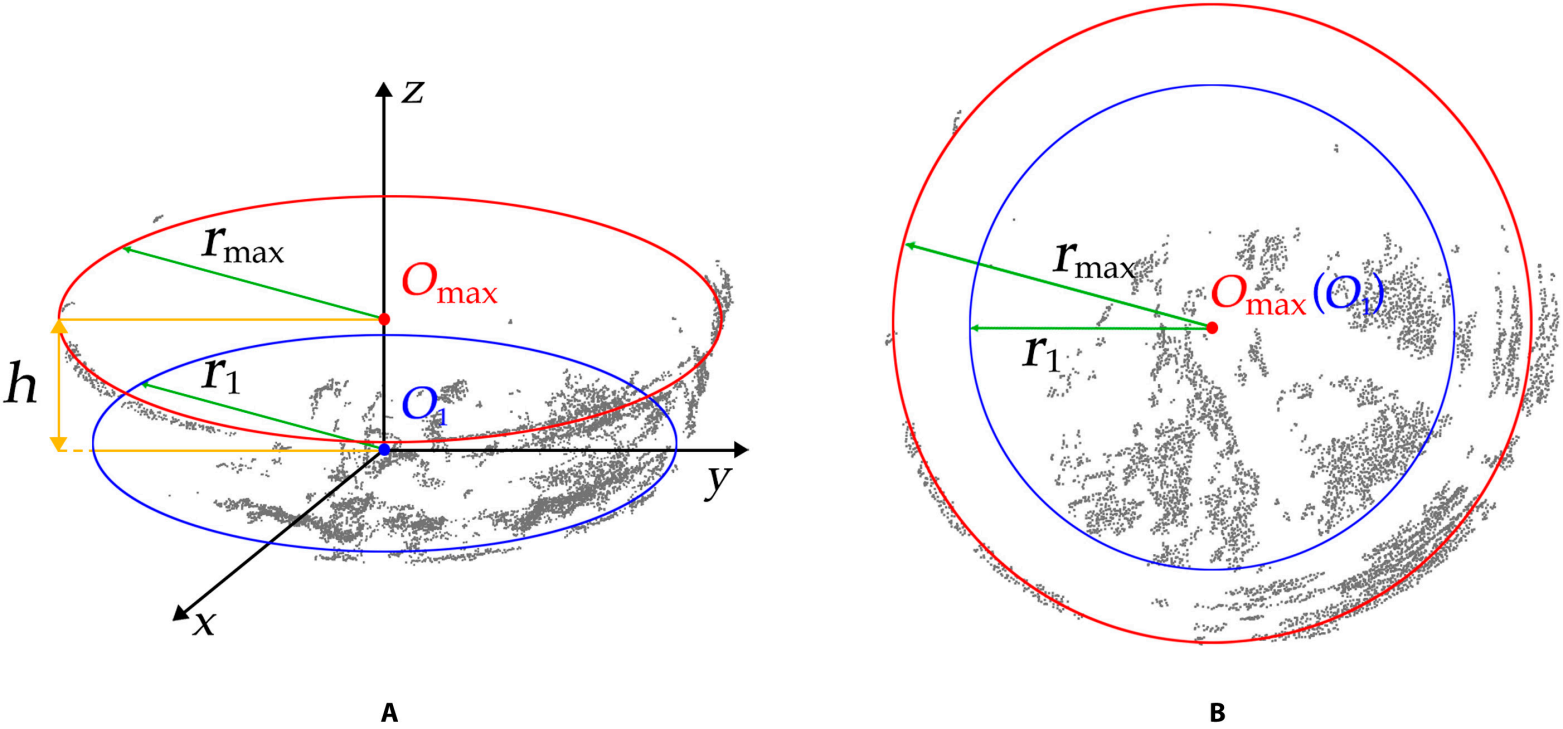
The pot point cloud and fitting circles, including (A) the front view and (B) the top view. *r*_max_ is the maximum radius of the fitting circle with center point *O*_max_. *r*_1_ is the radius of the bottom circle with center point *O*_1_. *h* is the distance between fitting circles.

As we only focused on plant points for subsequent analysis, the data preprocessing was first implemented by removing the noise and pot points within the raw data (Fig. 3B). The skeleton point extraction, connection, and skeleton optimization were then performed using SHS, followed by the segmentation of siliques. Finally, silique morphological parameters such as SN, SL, and SW were extracted based on the segmentation results. The overall workflow is shown in Fig. 2.

#### Preprocessing

Preprocessing of point clouds was performed to remove outlier noise points using statistical filtering [23,24]. Because the distribution of the pot points approximated an inverted frustum of a cone, the least-squares method (LSM) was employed to fit the circle [25]. On the basis of the fitting circle with the maximum radius found by LSM, we removed the points from up to the bottom inside the fitting circles. In Fig. 3B and C, *O*_max_ and *O*_1_ are the center points of the top and bottom fitting circles of the pot, respectively, while *r*_max_ and *r*_1_ are the corresponding radii. *h* is the distance between the top and bottom as described in Eq. 1:

$$h=z_{O_{\max}}-z_{O_{1}}$$ (1)

The fitting processing began from the bottom point *P*_1_(*x*_1_, *y*_1_, *z*_min_), which had the smallest *z* value. The points inside the range of [*z*_min_, *z*_min_ + ∆*d*] (∆*d* was the step distance) were projected into the plane *xP*_1_*y*, and the point *O*_1_ was calculated by LSM. This procedure was then repeated in the range of [*z*_min_ + (*n* − 1)*∆*d*, *z*_min_ + n*∆*d*] to obtain the fitting circle points *O_n_* (*n* = 1,2,3,…) until the largest fitting circle center point *O*_max_ and radius *r*_max_ were found. The pot data in the range of [*z*_min_, *z*_min_ + *h*] were then removed.

#### Skeleton point extraction

The L1-median point was defined as any point that minimized the sum of Euclidean distances to all points in the dataset. It had a good antinoise performance, and the L1-median point cloud could be set as the only global center of a given point set [26]. In this study, we took the L1-median point as a local skeleton point. For a given local point set *J* = {*p_i_*} with *n* points, *j* = 1,2,3,…,*n*, *J* ⊂ *R*^3^, the weighted center point of these points was computed iteratively by Eq. 2 to obtain the L1-median point *p*_*L*1_.

$$p_{L1}=A\left(J\right)=\sum_{j\in J}\frac{p_{j}w_{j}}{\sum_{j\in J}w_{j}\;}$$ (2)

where *w_j_* is the weight factor. As the L1-median points could present a sparse distribution where the local center points were excessively clustered together [27,28], the conditional regularization was used to shift each point into its respective geometric center position to avoid sparse distribution [18,27,28]. A repulsive force was applied between the L1-median points to keep these points at an appropriate distance from each other. The position of each skeleton point was then calculated by attractive item A(*J*) and repulsive item R(*I*) (Eq. 3). The Gaussian kernel function *θ*(∙) and the distance function ‖∙‖ were used to give the weights of neighborhood points (Eqs. 4 and 5) so that the impact of random and uneven distribution of points could be reduced.

$$p_{L1}^{k+1}=A\left(J\right)+R\left(I\right)=\frac{\sum_{j\in J}p_{j}\alpha_{j}^{k}}{\sum_{j\in J}\alpha_{j}^{k}}+\tau \lambda_{i}^{k}\frac{\sum_{i\in I}\left(p_{L1}^{k}-p_{i}^{k}\right)\beta_{i}^{k}}{\sum_{i^{\prime }\in I}\beta_{i}^{k}\;}$$ (3)

$$\alpha_{j}^{\kappa }=\frac{\theta \left(\left\|p_{L1}^{k}-p_{j}\right\|\right)}{\left\|p_{L1}^{k}-p_{j}\right\|},j=1,2,3,\ldots ,n,p_{j}\in J$$ (4)

$$\beta_{i}^{k}=\frac{\theta \left(\left\|p_{L1}^{k}-p_{i}^{k}\right\|\right)}{{\left\|p_{L1}^{k}-p_{i}^{k}\right\|}^{2}},i=1,2,3,\ldots ,m,p_{i}^{k}\in I$$ (5)

where $p_{L1}^{k+1}$ is the selected L1-median point, and *k* is the number of iterations. *J* is the source neighborhood points of $p_{L1}^{k+1}$, while *I* is the neighborhood L1-median points of $p_{L1}^{k+1}$. $\;\alpha_{j}^{\kappa }$ is the attractive factor, while $\beta_{i}^{k}$ is the repulsion factor. *τ* is a factor for balancing attraction and repulsion. $\lambda_{i}^{k}$ is the directionality degree of $p_{L1}^{k}$, which is used to give the repulsion direction.

**Fig. 4.**
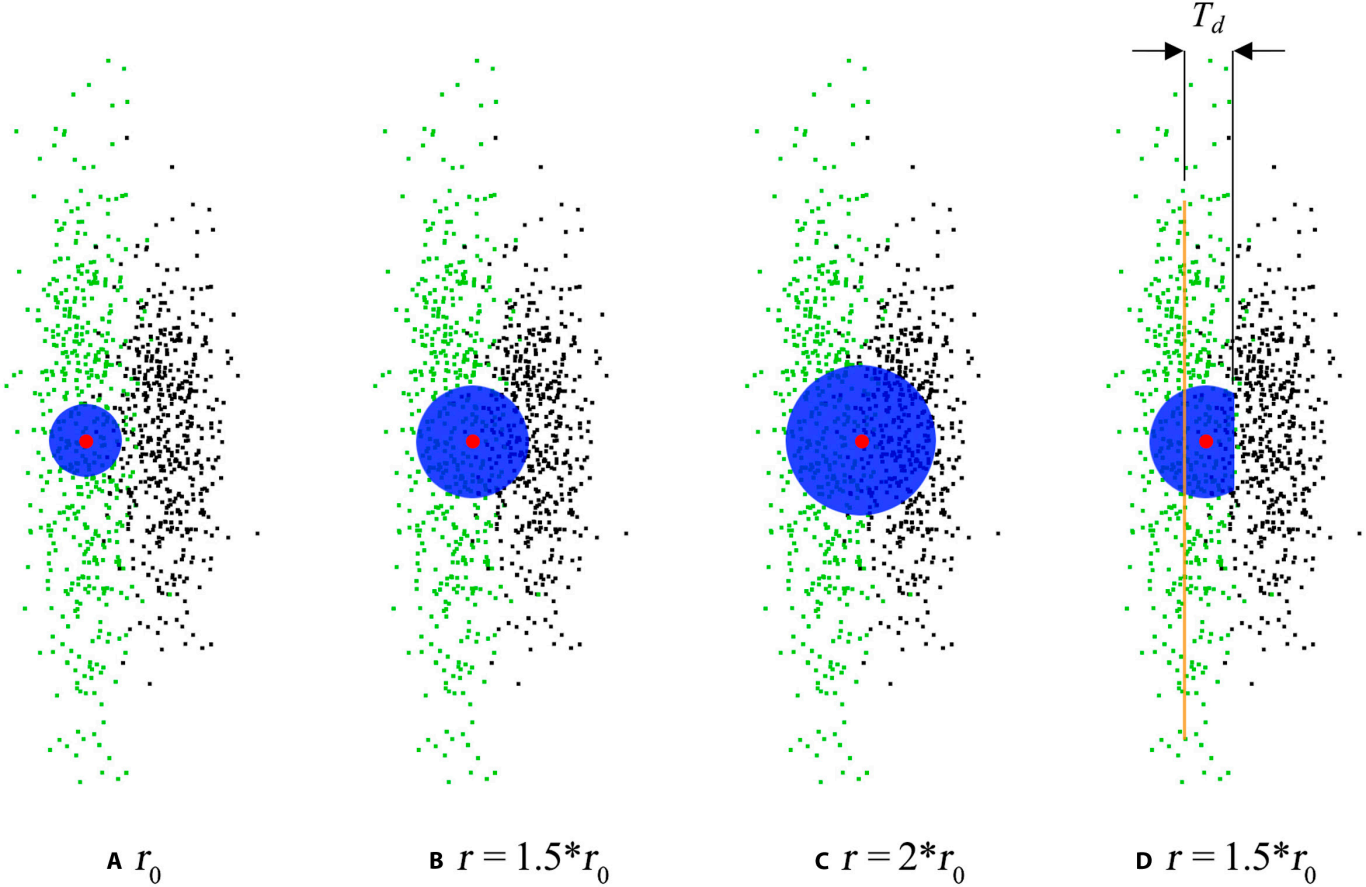
L1-median points of local regions with different radiuses. *r*_0_ is the initial radius. The red dot is the L1-median point. The blue circle is the radius of the local region, and green and black points represent 2 different objects. The yellow line represents the RANSAC fitting plane. *T_d_* is the limited distance threshold.

In each iteration, the radius of the neighborhood was increased to avoid the position deviation of the L1-median point (red dot). However, the increased radius (blue circle) could introduce interferential points (black points) from other objects, which were not in the same object as the green points and thus caused its position deflection (Fig. 4A to C). To avoid interference of the points from different objects, the calculating range was limited by the fitting plane ∏*_R_* (Fig. 4D). Because of the flat and long shape of a silique, we applied the random sample consensus algorithm (RANSAC) for detecting the fitting plane ∏*_R_* [29]. Only the points whose Euclidean distance from plane ∏*_R_* was less than the limited distance threshold *T_d_* were used to calculate the skeleton point’s position. The neighborhood points were updated by Eqs. 6 and 7:

$$J_{R}=\left\{p_{j}|∥p_{j},\prod_{R}∥<T_{d},p_{j}\in J\right\}$$ (6)

$$I_{R}=\left\{p_{i}|∥p_{i},\prod_{R}∥<T_{d},p_{i}\in I\right\}$$ (7)

where *J*_R_ is the point set constrained by ∏*_R_* from source *J*. *I*_R_ is the point set constrained by ∏*_R_* from *I*. ‖*p_j_*, ∏*_R_*‖ and ‖*p_i_*, ∏*_R_*‖ are the distances between the points and ∏*_R_*.

#### Skeleton point connection

Because there were no topological relationships between discrete skeleton points, it was necessary to connect the skeleton points for silique segmentation. The local partition and growing algorithm were used to connect the skeleton points. The density-based spatial clustering of applications with noise (DBSCAN) performed well in finding various shapes [30], and it was first employed to roughly cluster skeleton points into several classes. A graph theoretical algorithm was proposed to connect the points in the same class. Here, we used the weighted unidirectional graph (WUG) to connect the skeleton points of the same class. WUG was used to solve the shortest-path problem, and it built connections between the skeleton points after giving an initial searching direction with the Euclidean distance as the weight factor. In general, the tangent vector of a skeleton point can represent the extension or searching direction. Because the skeleton changes smoothly and the position of the initial point *p_i_* within the class is random, the tangent vector function can be simplified as Eq. 8. If *p_i_*(*x_i_*, *y_i_*, *z_i_*) is the end point, then *p_i_* and the nearest point *p*_*i*−1_ (*x*_*i*−1_, *y*_*i*−1_, *z*_*i*−1_) are used to get a tangent vector. Otherwise, the nearest 2 points *p*_*i*−1_ (*x*_*i*−1_, *y*_*i*−1_, *z*_*i*−1_) and *p*_*i*+1_ (*x*_*i*+1_, *y*_*i*+1_, *z*_*i*+1_) are used. We set the initial direction of the initial point to be positive.

$$\left\{\begin{array}{c} \frac{x-x_{i-1}}{x_{i}-x_{i-1}}=\frac{y-y_{i-1}}{y_{i}-y_{i-1}}=\frac{z-z_{i-1}}{z_{i}-z_{i-1}}\;\mathrm{if}\;p_{i}=\text{end point}\\ \frac{x-x_{i-1}}{x_{i+1}-x_{i-1}}=\frac{y-y_{i-1}}{y_{i+1}-y_{i-1}}=\frac{z-z_{i-1}}{z_{i+1}-z_{i-1}}\;\mathrm{if}\;p_{i}\neq \text{end point} \end{array}\right.$$ (8)

**Fig. 5.**
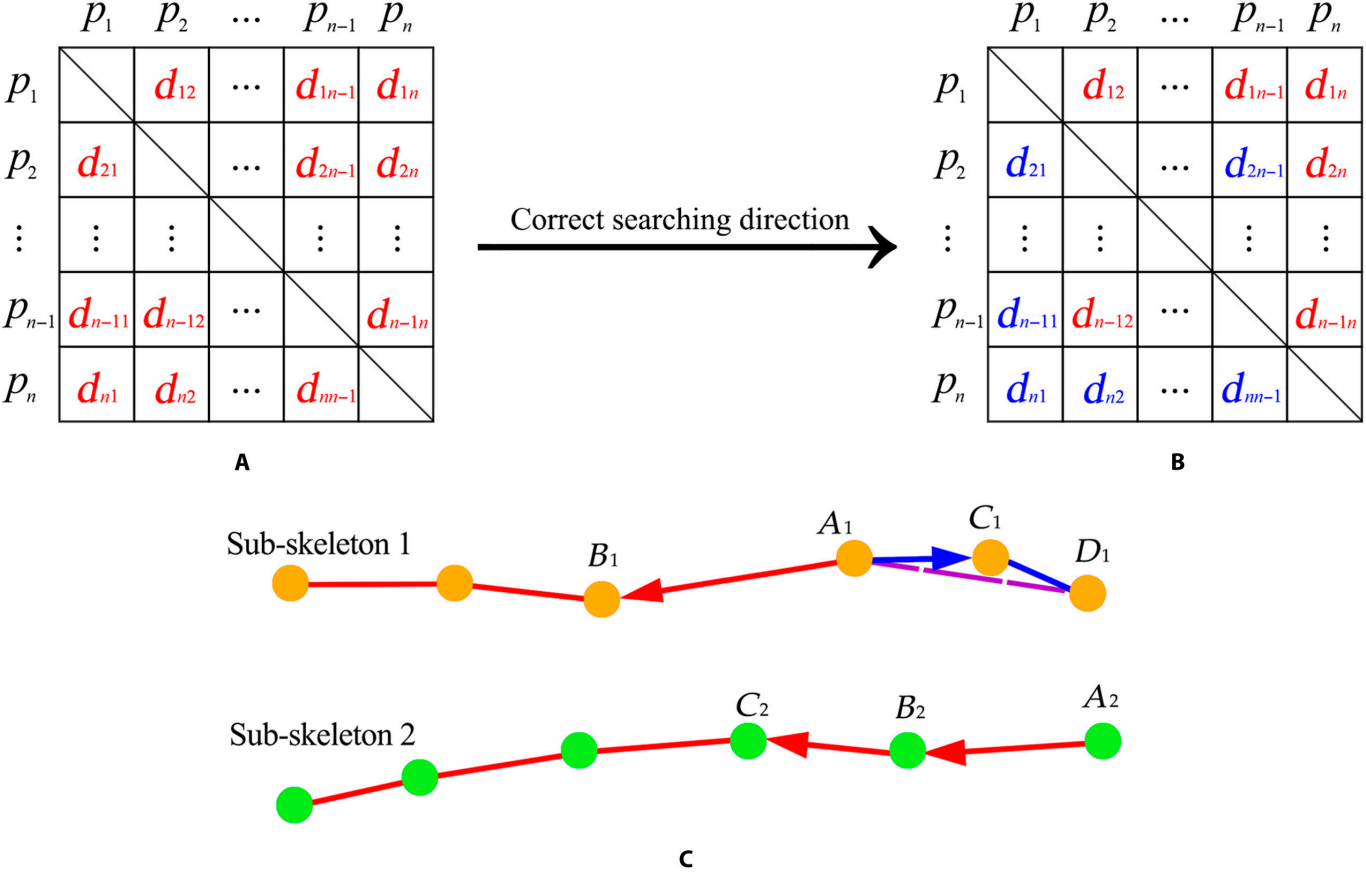
(A) The adjacency list without the given initial direction, and all the weights are positive (red). (B) The adjacency list with the given initial direction, and the weights are negative (blue). *p*_1_, *p*_2_, …, *p_n_* are the skeleton points. *d_ij_*, *i*,*j*=1, 2, …, *n*, *i*≠*j*, is the Euclidean distance weight factor. (C) The connection of skeleton points of different organ skeletons by DBSCAN and WUG. Sub-skeleton 1 and sub-skeleton 2 are 2 classes. The red lines indicate that the weights are positive, while the blue lines indicate that the weights were negative. In sub-skeleton 1, *A*_1_ is the non-end point, and WUG search directions are $\vec{A_{1}C_{1}}$ and $\vec{A_{1}B_{1}}$; *A*_1_*D*_1_ (purple dotted line) is the wrong connection line. In sub-skeleton 2, *A*_2_ is the end point, and the WUG search directions are $\vec{A_{2}B_{2}}\;$and $\vec{B_{2}C_{2}}$.

For skeleton points in the same class, the WUG stores the weight factor between 2 points in the form of an adjacency list. Without correcting the searching direction, all the weights are positive, with *d_ij_* = *d_ji_* (Fig. 5A), indicating that the connection of skeleton points depends on the absolute value of distance, which may cause the wrong connection lines of skeleton points. For example, if weight *d*_*A*_1_*D*_1__ is smaller than *d*_*A*_1_*B*_1__ in sub-skeleton 1 (Fig. 5C), then *A*_1_ would be wrongly connected with *D*_1_. Therefore, the searching directions of each point are corrected by previous connecting points, and some weights in the adjacency list changed to be negative (Fig. 5B). We defined the out-degree (OD) and in-degree (ID) to limit the searching direction. Given a point *p*, OD is the number of lines coming out of *p*, while ID is the number of lines coming into *p*. End points only have one edge, indicating OD = 1 or ID = 1. For non-end points, we limited the points to have only 2 edges, indicating OD = 1 and ID = 1. As shown in Fig. 5C, sub-skeleton 1 with yellow points and sub-skeleton 2 with green points are 2 classes divided by DBSCAN. They are adjacent in the 3D space, and the WUG searches only the points within the same class. In sub-skeleton 1, the initial point *A*_1_ is a non-end point and only connects the nearest 2 points, *B*_1_ and *C*_1_. If the initial point is an end point such as *A*_2_ in sub-skeleton 2, then it would only connect to the nearest point *B*_2_, and then *C*_2_ would be connected by *B*_2_.

#### Skeleton optimization

**Fig. 6.**
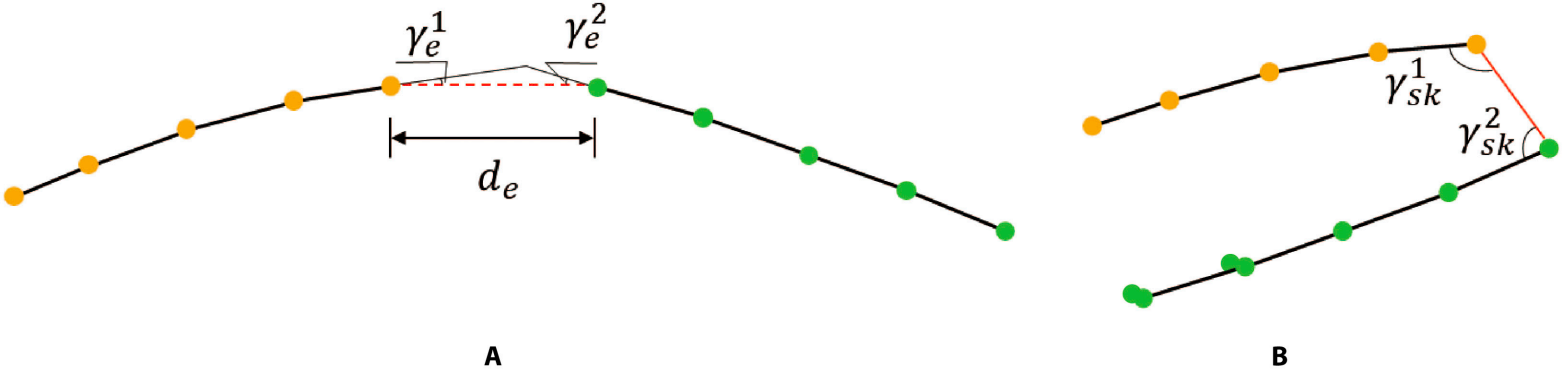
Two incorrect cases after skeletonization. (A) One silique or stem is clustered into 2 classes (yellow and green), and the red dashed line is the wrong unconnected line. (B) Two siliques or stems (yellow and green) are clustered into one class, and the red solid line is the wrong connected line. $\gamma_{e}^{1}$ and $\gamma_{e}^{2}$ are the angles between end points’ tangent vectors and end points’ connection lines. *d_e_* is the distance between 2 end points from different classes. $\gamma_{\mathrm{sk}}^{1}$ and $\gamma_{\mathrm{sk}}^{2}$ are the angles between each point’s 2 edges.

Some silique skeletons were still incorrectly connected because of the limited clustering performance of DBSCAN. These incorrect cases would reduce the accuracy of silique segmentation. As shown in Fig. 6A, one silique or stem is clustered into 2 classes (yellow and green). Because the skeleton changes smoothly, the distance (*d_e_*) between end points and the angles ($\gamma_{e}^{1}$, $\gamma_{e}^{2}$) between end points’ tangent vectors and end points’ connection lines (red dotted) are small. Thus, we calculated the average distance ($d_{\bar{\mathrm{sk}}}$) between connected skeleton points and connected the 2 adjacent classes that met the following 2 requirements: (a) Both $\gamma_{e}^{1}$ and $\gamma_{e}^{2}$ were smaller than 15°; (b) *d_e_* was smaller than 5 $\times d_{\bar{\mathrm{sk}}}$. In addition, different siliques could also be clustered into the same class. Thus, we set the angles ($\gamma_{\mathrm{sk}}^{1}$, $\gamma_{\mathrm{sk}}^{2}$) between each point’s 2 edges larger than 165° to divide nearby siliques into different classes; only if $\gamma_{\mathrm{sk}}^{1}$ and $\gamma_{\mathrm{sk}}^{2}$ were both larger than 165°, then the sub-skeletons were considered as the same silique class (Fig. 6B).

#### Silique segmentation and morphological parameter extraction

After skeletonization and optimization, each silique or stem had its own unique corresponding sub-skeleton, and the skeleton points were ordered under the given direction in the sub-skeletons. On the basis of the assumption of the normal distribution or near-normal distribution of SL [6,31,32], we used siliques with SL within the 95% confidence interval as the effective siliques (SL*_ef_*) and counted the SN. Additionally, silique abortion was a normal phenomenon for oilseed rape [33], and the stunted siliques were tenuous or small in shape and without seeds. Thus, siliques shorter than 15 mm were removed. Here, the sub-skeleton length *l*_{*b_j_*}_ was calculated as the sum of the distances of all adjacent skeleton points in this sub-skeleton {*b_j_*} (Eq. 9). Because of the influence of extreme values such as long stems, only 60% of the middle length data was used to calculate the mean value *μ* of the normal distribution of SL (Eq. 10). After removing aborted siliques, SL*_ef_* was defined as Eq. 11. The total SL was the sum of *SL_ef_* (Eq. 12).

$$l_{\left\{b_{j}\right\}}=\left\{\sum \left\|p_{i}-p_{i+1}\right\|,p_{i}\in \left\{b_{j}\right\},i=1,2,3\ldots m-1;j=1,2,3,\ldots ,n\right\}$$ (9)

$$\mu =\frac{\sum_{j=\mathrm{int}\left(0.2m\right)}^{0.8m}l_{\left\{b_{j}\right\}}}{0.6m}$$ (10)

$${\mathrm{SL}}_{\mathrm{ef}}\in \left[\text{max}\left(15\;\mathrm{mm},\mu -1.96*\frac{\sigma }{\sqrt{n}}\right),\mu -1.96*\frac{\sigma }{\sqrt{n}}\right]$$ (11)

$$\text{Total}\;\mathrm{SL}=\sum_{i=0}^{m^{\prime }}{\mathrm{SL}}_{\mathrm{ef}}$$ (12)

where *m* is the number of skeleton points in the sub-skeleton, and *p_i_* is a skeleton point. ‖*p_i_* − *p*_*i*+1_‖ represents the Euclidean distance between *p_i_* and *p*_*i*+1_. *σ* is the standard deviation (SD). *m*^′^ is the number of effective siliques.

**Fig. 7.**
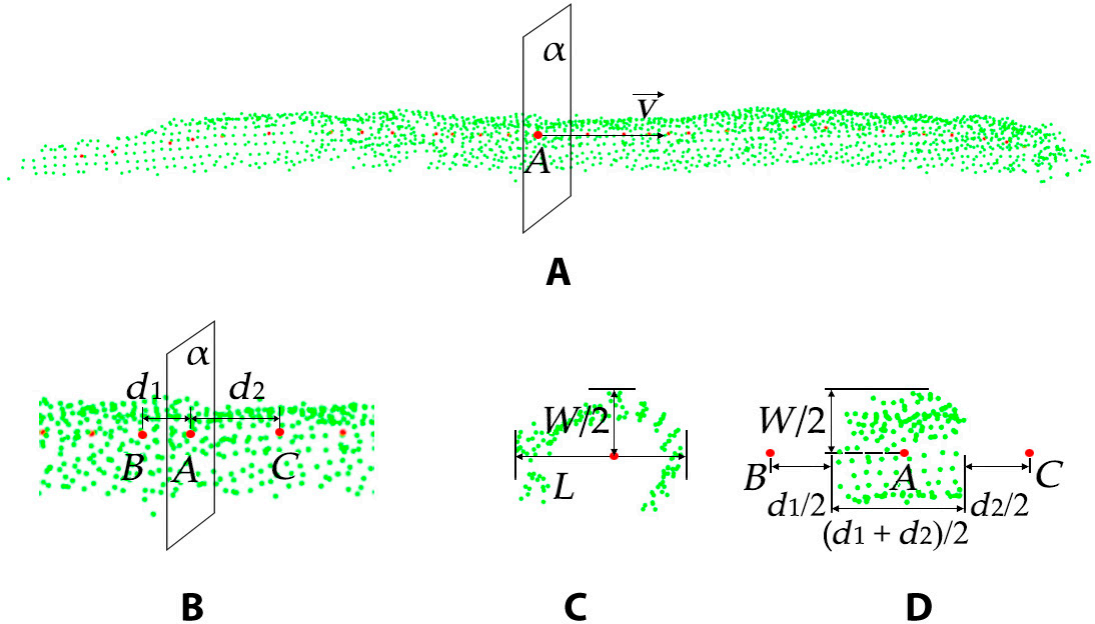
The process of volume calculation. (A) Source point (green) cloud and skeleton points (red). (B) Partial data around point *A*. (C) The distribution of projected neighborhood points of *A*. (D) Points inside the space that point *A* represents. Above all, *A*, *B*, and *C* are skeleton points. $\vec{v}$ is the direction of skeleton point *A*, and plane *α* is the normal plane of *A*. *d*_1_is the distance between *A* and *B*, while *d*_2_ is the distance between *A* and *C*. *W* is the width, and *L* is the length.

Furthermore, the classified silique points were obtained by searching the neighborhood source points of the RANSAC constraint plane, and the silique volume (SV) was calculated based on the silique points. As shown in Fig. 7, the green points are the source points of the siliques, and the red points are the skeleton points. In Fig. 7A and B, $\vec{v}$ is the direction of a skeleton point *A*, and plane *α* is the normal plane of *A*. Points *B* and *C* are the adjacent skeleton points of *A* (Fig. 7B and D). *d*_1_ and *d*_2_ are the corresponding distances. The source points inside the range of [*A* − *d*_1_/2, *A* + *d*_2_/2] were projected onto plane *α* (Fig. 7C). Assuming that the distribution of the projected points was rectangular, *W* and *L* were the width and length of the rectangle, and the partial volume of skeleton point *A* was calculated (Eq. 13). All the partial volumes were added to obtain a single silique (Eq. 14). Similarly, we also calculated the total SV by Eq. 15:

$$V_{A}=L*W*\frac{d_{1}+d_{2}}{2}$$ (13)

$$\mathrm{SV}=\sum_{i=1}^{n}L_{i}*W_{i}*\frac{d_{1}^{i}+d_{2}^{i}}{2}$$ (14)

$$\text{Total}\;\mathrm{SV}=\sum_{j=1}^{m^{\prime }}V_{j}$$ (15)

where *n* is the skeleton point number and *m*^′^ is the number of effective siliques.

### Validation and statistical analysis

The architecture of oilseed rape was related to the branch. We denoted 3 types of branches as follows: the first branch (*B*_1st_), also called the main stem; the second branch (*B*_2nd_), grown on the first branch; and the third branch (*B*_3rd_), grown on the second branch [34].

To validate the performance of the SHS algorithm, the recall (*Re_EL_*) was computed by Eq. 16, representing the proportion of estimated siliques (SN*_E_*) to siliques counted by the laser point cloud (SN*_L_*). In addition, the SN manually measured (SN*_M_*) was taken as the ground truth, and the recall (*Re_LM_*) was computed by Eq. 17 to evaluate the quality of the laser-scanned point cloud, representing the ratio of SN*_L_* to SN*_M_*. The recall (*Re_EM_*) computed by Eq. 18 represents the ratio of SN*_E_* to SN*_M_*.

ReEL=SNESNL×100% (16)

ReLM=SNLSNM×100% (17)

ReEM=SNESNM×100% (18)

Additionally, to test the correlation between SHS estimations and manual measurements, the coefficient of determination (*R*^2^) and the root mean squared errors (RMSE) were calculated. Because mature siliques were easily broken during the transportation of oilseed rape, measuring the length of these siliques was difficult. Therefore, we used the total SL of a single plant grown in the greenhouse to verify the accuracy of SL estimation. According to linear regression analyses, we used the correlation coefficient *R* to explore the relationship between SL, SN, and SV and the yield of a plant (YOP), where YOP was denoted as the total SW in siliques.

The proposed algorithm was developed by Point Cloud Library [35] and Open3D Library [36] in the Visual Studio 2015 and PyCharm 2011 development platforms. The algorithm was implemented on a desktop workstation with the configuration of an Intel Core i7 processor and 16 GB of memory.

## Results

### The visualization of plant architecture

**Fig. 8.**
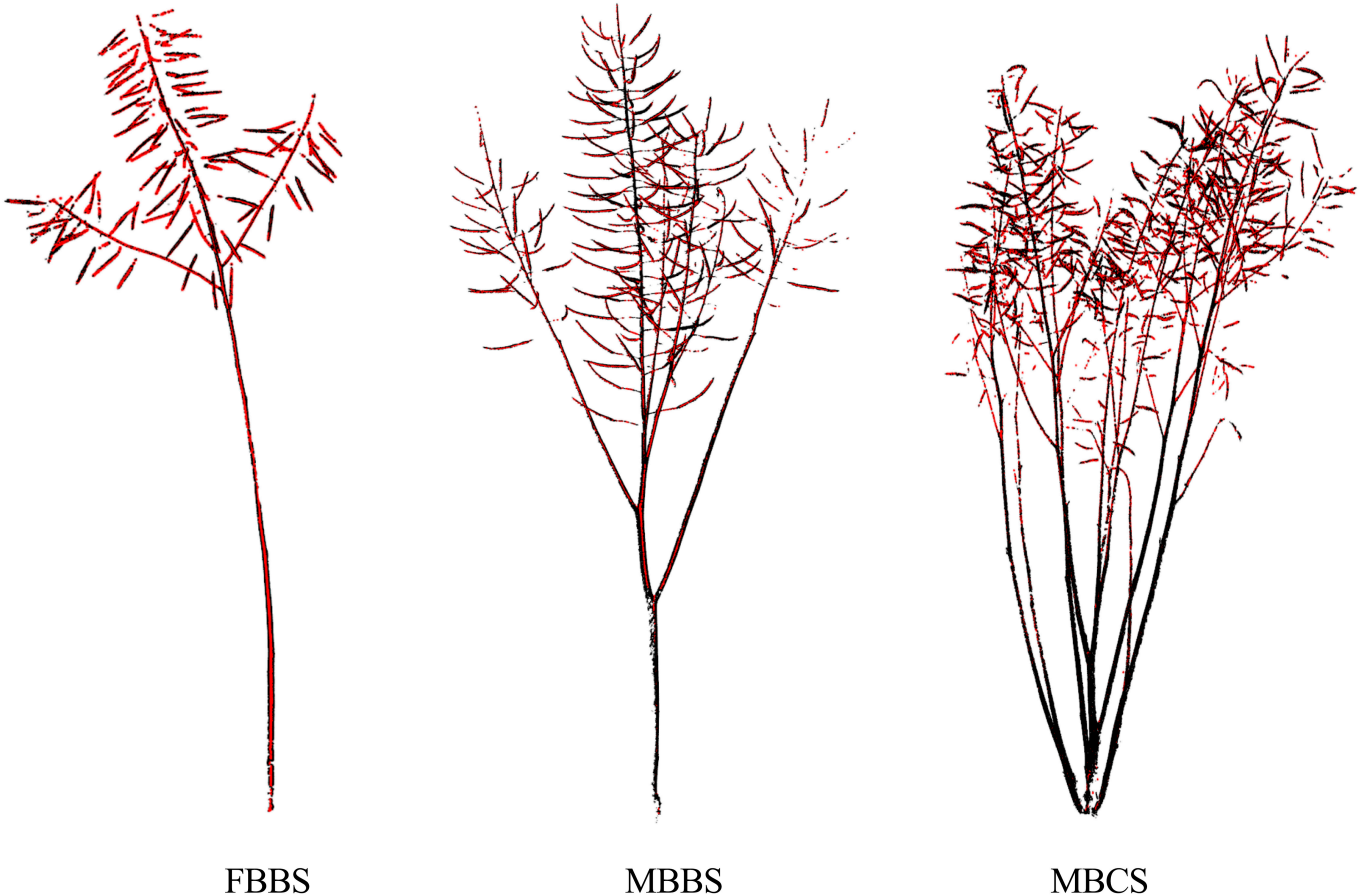
Examples of source point clouds and skeleton points (red dots) of 3 plant architectures of oilseed rape. FBBS, MBBS, and MBCS represent the few-branch broom shape, the multibranch broom shape, and the multibranch cylinder shape, respectively.

Figure 8 presents 3 typical plant architectures of oilseed rapes. There were obvious differences among the 3 types. The plants of FBBS only had one *B*_1st_ and few or no *B*_2nd_s and *B*_3rd_s, and the canopy was sparse. The plants of MBBS also only had 1 or 2 *B*_1st_s but more *B*_2nd_s and *B*_3rd_s with a dense canopy. Compared with FBBS and MBBS, the plants of MBCS had more *B*_1st_s with multiple second and third branches. The silique canopy was evenly distributed and cylindrical.

### Siliques segmentation

**Fig. 9.**
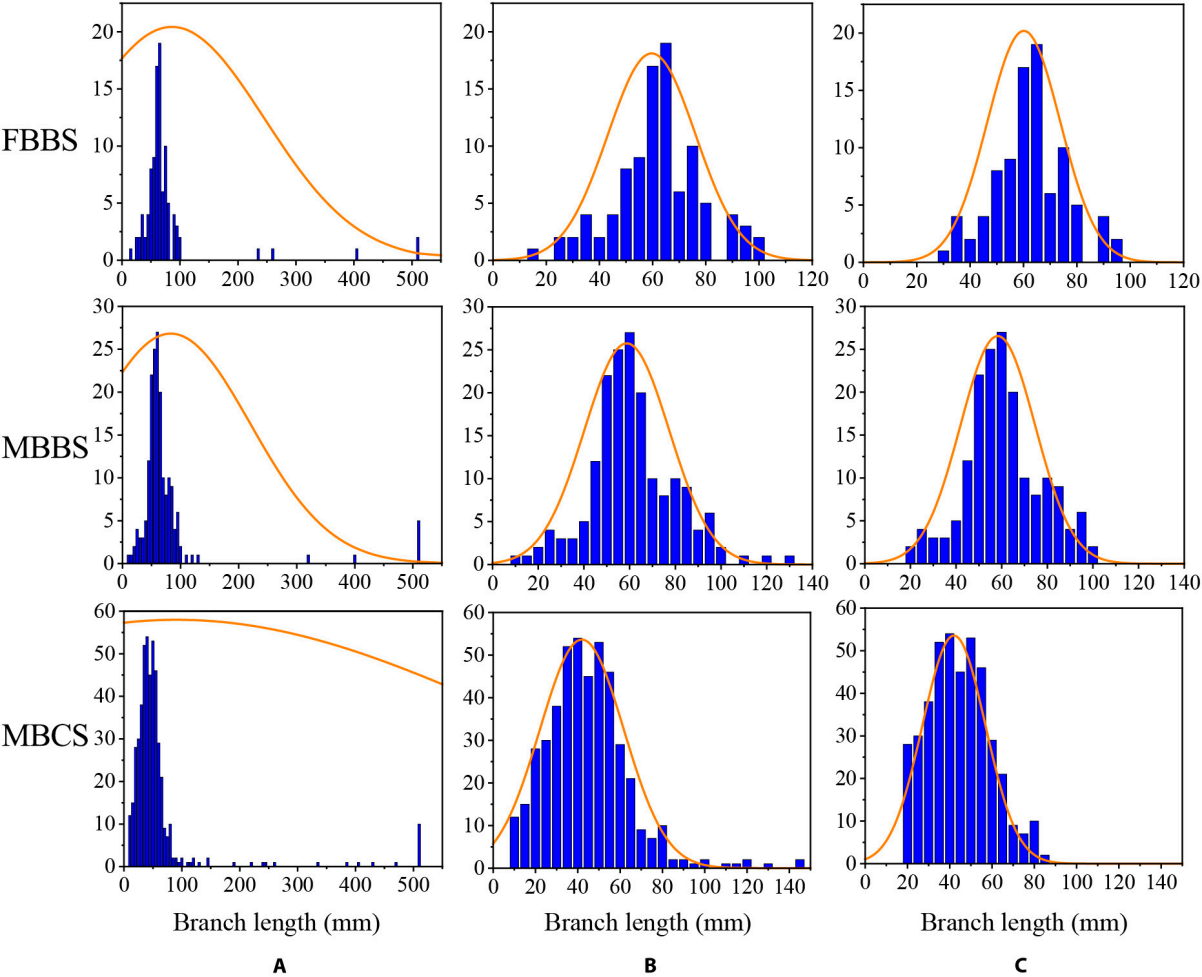
Histograms of the sub-skeleton length of 3 plant architectures. FBBS, MBBS, and MBCS represent the few-branch broom shape, the multibranch broom shape, and the multibranch cylinder shape, respectively. (A) The length distribution of all sub-skeletons, including branches and siliques, and the sub-skeletons longer than 500 mm were combined into one group for display convenience. (B) The length distribution of sub-skeletons without long stems. (C) The length distribution of effective siliques.

**Fig. 10.**
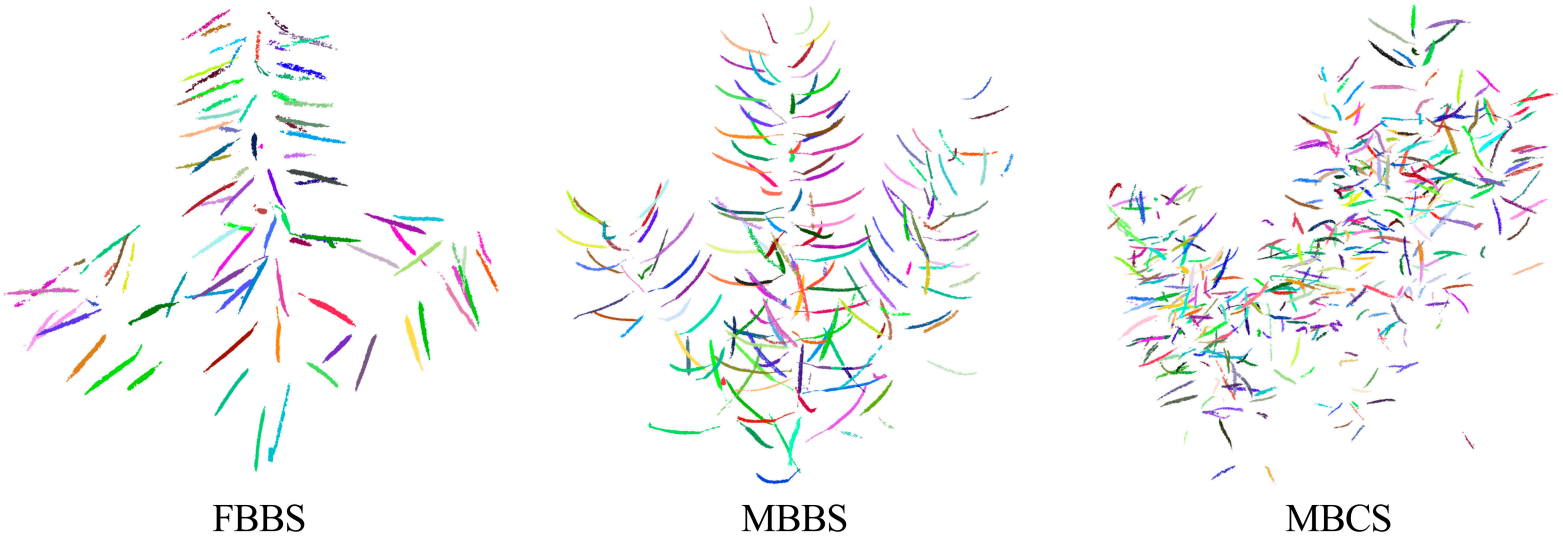
The segmented and classified siliques point cloud of 3 plant architectures. Each silique was rendered by a unique color. The FBBS, MBBS, and MBCS represented 3 plant architectures: the few-branch broom shape, the multibranch broom shape, and the multibranch cylinder shape, respectively.

We counted the lengths of sub-skeletons of the 3 plant architectures, and the sub-skeletons included stems/branches and siliques. Through manual verification, 95% of the sub-skeletons over 200 mm were long branches, which was significantly different from that of siliques (Fig. 9A). After removing the branch sub-skeleton, the length distribution of all sub-skeletons of a single plant followed the assumption of a normal distribution or near-normal distribution (Fig. 9B). In addition, the aborted siliques without seeds were removed. Finally, we obtained the effective siliques in a 95% confidence interval of length distribution, which also followed the above assumption (Fig. 9C). At the same time, these results proved that our materials were conventional, and the results were universal. The effective siliques were rendered by different colors (Fig. 10).

On the basis of the differences in branches, we obtained 13, 6, and 5 plants of FBBS, MBBS, and MBCS, respectively. There were significant differences between these 3 plant architectures (Table 2). The plants of FBBS only had one average *B*_1st_, 3.69 average *B*_2nd_s, and almost no *B*_3rd_s. There were more second and third branches in the MBBS plants, which had 6.33 average *B*_2nd_s and 4.33 *B*_3rd_s. The numbers of the first, second, and third branches of MBCS plants was the highest, with the average values of 4.20, 14.20, and 6.20, respectively. In addition, the average SN of MBCS plants were the largest at 549.60, while those of MBBS and FBBS were 316.17 and 115.54, respectively. More details about the siliques and branches of each plant are presented in the Supplementary Materials.

**Table 2.** Segmentation results of siliques with different plant architectures.

Plant architecture		*NB*_1st_	*NB*_2nd_	*NB*_3rd_	SN*_M_*	SN*_L_*	SN*_E_*	*Re_LM_* (%)	*Re_EL_* (%)	*Re_EM_* (%)
FBBS	Average	1	3.69	0.08	115.54	108.08	102.23	94.97	93.91	82.50
	SD	0	1.64	0.27	65.61	60.23	58.81	5.85	4.85	7.42
MBBS	Average	1.33	6.33	4.33	316.17	295.33	265.00	93.45	90.27	85.43
	SD	0.47	0.75	2.29	114.06	100.50	88.89	3.69	3.80	6.13
MBCS	Average	4.20	14.20	6.20	549.60	446.8	374.00	82.33	83.81	69.22
	SD	1.17	3.66	2.14	93.80	76.41	63.83	12.02	4.86	12.38
Average	/	/	/	/	/	/	/	92.23	90.90	84.23
SD	/	/	/	/	/	/	/	8.82	6.07	11.54

SN*_M_*, silique number measured manually; SN*_L_*, silique number counted by laser point cloud; SN*_E_*, silique number estimated by our method; SD, standard deviation; *Re_LM_*, recall = SN*_L_*/SN*_M_*; *Re_EL_*, recall = SN*_E_*/SN*_L_*; *Re_EM_*, recall = SN*_E_*/SN*_M_*; *NB*_1st_, the number of first branches; *NB*_2nd_, the number of second branches; *NB*_3rd_, the number of the third branches.

**Fig. 11.**
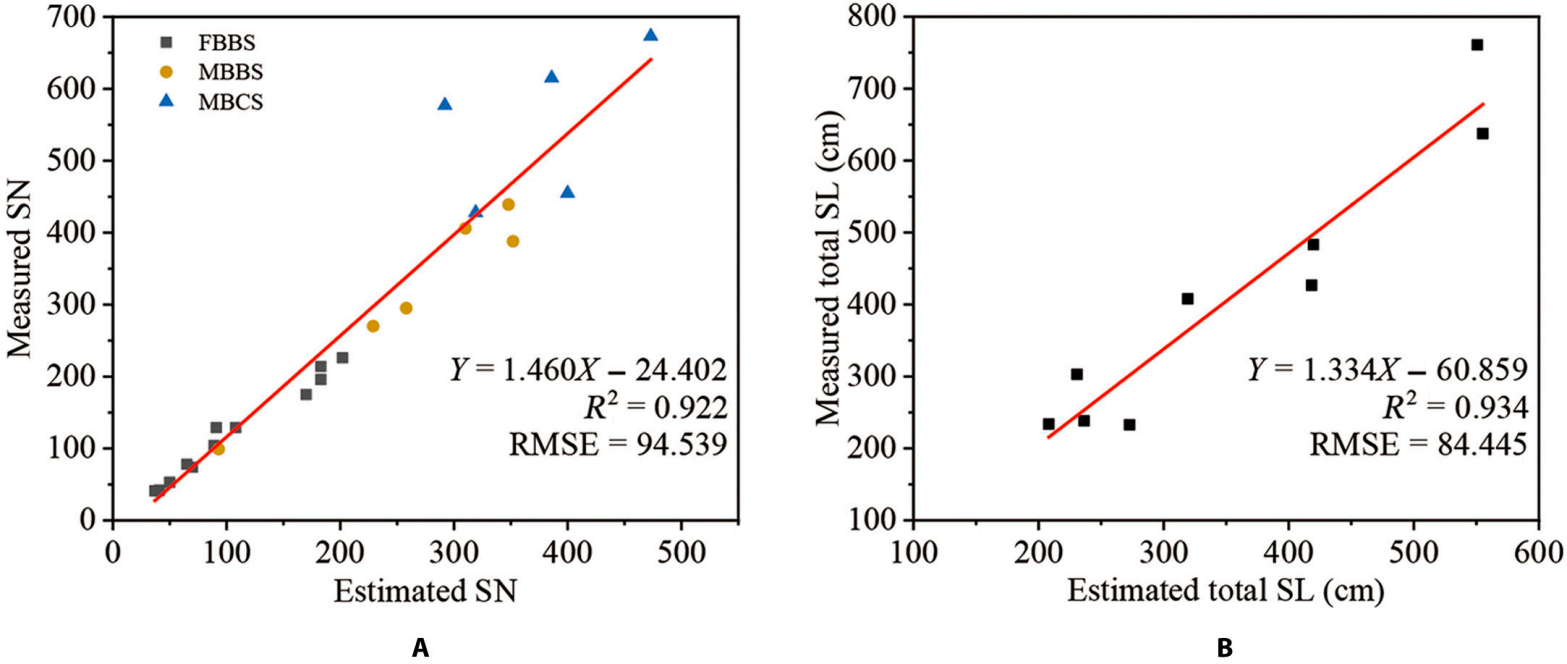
The relationship between morphological parameters derived by manual measurement and the SHS. The morphological parameters include SN and total SL. (A) The relationship of SN of all cultivars, including 3 plant architectures: FBBS, MBBS, and MBCS. (B) Comparison of the total SL of cultivars (c1 to c3) grown in a greenhouse.

Because of the SHS algorithm being carried on the laser point cloud, the estimation of the SN was affected by the data quality of the point cloud and the performance of the algorithm. Therefore, the results were evaluated from these 2 perspectives. For all cultivars, the average recall (*Re_LM_*) between SN*_L_* and SN*_M_* was at a high level of 92.23% with an SD of 8.82, indicating that most of the plants were well scanned, and the laser point cloud of siliques was complete. The average recall (*Re_EL_*) between SN*_E_* and SN*_L_* was 90.90% with a low SD of 6.07, indicating the accurate and stable performance of the SHS algorithm. On the basis of the data of good quality and algorithm performance, the SN*_E_* was well correlated with SN*_M_*, and the average recall (*Re_EM_*) between SN*_E_* and SN*_M_* was 84.23% with an *R*^2^ of 0.922 (Fig. 11A).

Moreover, the structure of plants affected the data quality and performance of the algorithm. The plants of FBBS and MBBS architectures were well scanned with the point cloud of high completeness, and the siliques were well-segmented, so the average *Re_LM_*s were of high value (FBBS was 94.47%; MBBS was 93.45%), as well as average *Re_EL_*s (FBBS was 93.91%; MBBS was 90.27%). In contrast, the plants of MBCS architecture had significant differences, with more second branches, third branches, and siliques. Many silique data were missing in the scanning process, causing low *Re_LM_*s, which affected the data quality and the performance of the algorithm, decreasing the average *Re_EL_* to 83.81%. Additionally, the total SL was accurately estimated with an *R*^2^ of 0.934 (Fig. 11B)

### The relationship between silique morphological parameters and the yield of a plant

**Fig. 12.**
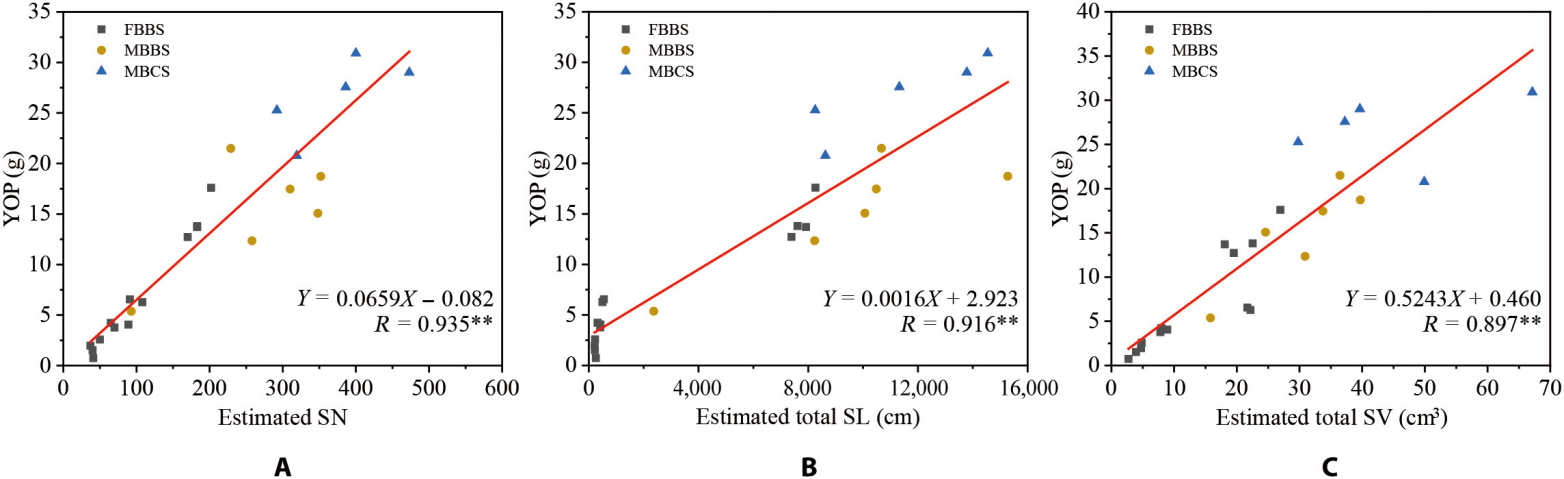
The relationship between the yield of a plant and estimated morphological parameters of all cultivars with 3 plant architectures, including the FBBS, the MBBS, and the MBCS. The parameters include SN (A), total SL (B), and total SV (C). ** indicates statistical significance at 0.01.

The differences in plant architecture*s* affected YOP (Fig. 12A to C). The structures of plants of FBBS and MBBS were similar, and the main difference was the branch number, so the YOP of MBBS was higher than that of FBBS. The structure of MBBS was significantly different and its yield was higher. The SN, total SL, and total SV estimated by our method were significantly positively correlated with YOP, and the *R* values were 0.935, 0.916, and 0.897, respectively*.*

## Discussion

### Acquisition of high-quality point cloud data

Currently, the phenotypic identification related to oilseed rape yield is still dependent on manual methods, which are time-consuming and labor-intensive, and the results are greatly influenced by subjective factors. In contrast, methods based on 3D imaging have the advantages of high automation and more objective results. Among them, the laser point cloud is accurate and reliable and has been widely applied for agricultural research, such as plant reconstruction and parameter extraction [21,22]. However, the laser point cloud scanning process is tedious. Because the plants are nonrigid objects that are irregularly deformed by the wind, the scanning process requires a windless environment, which greatly affects the acquisition efficiency. In addition, silique breakage and falling occurring during plant transfer and silique abortion also affect the results of morphological analysis, resulting in incorrect or missing data. Therefore, the optimization of point cloud data acquisition is essential. RGB-D cameras and stereo cameras have shown potential for field-based plant phenotyping [17,37,38], but these sensors have shortcomings in accuracy and stability. It is necessary to improve the resolution and accuracy of these sensors and develop corresponding 3D imaging algorithms for the acquisition performance. To achieve accurate, efficient, and invasive yield estimation in the field, a field phenotyping platform with integrated multiple sensors needs to be further investigated.

**Fig. 13.**
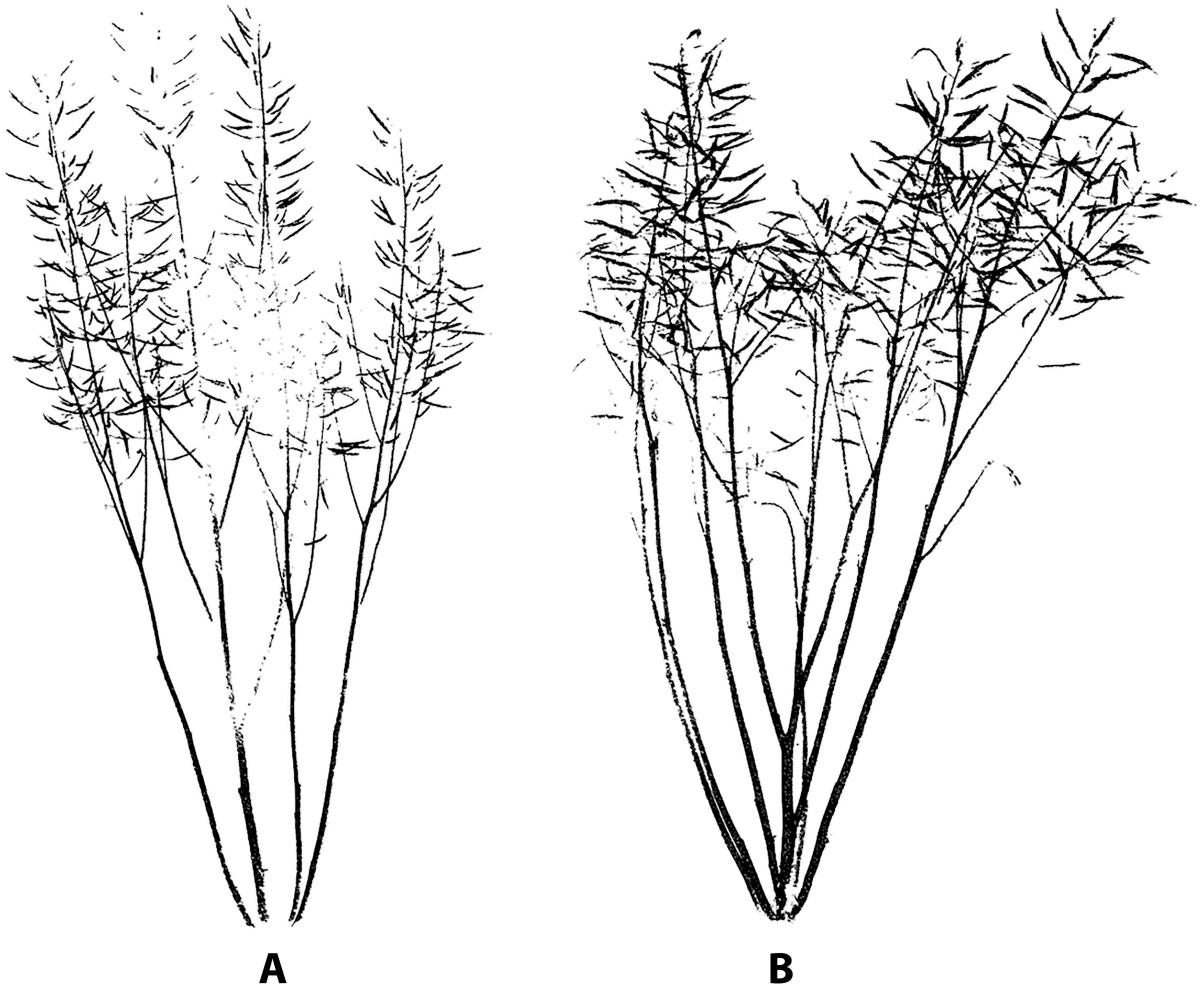
The plants of MBCS architecture. (A) CR3168 (c7)-1; (B) Hu135 (c8)-1.

Occlusion has an important effect on the quality of the point clouds. It is one of the main limitations of acquiring plant parameters by computer vision technology and is always accompanied by missing data. The probability of occlusion is different for plants with different structures, and complex structures are more likely to introduce the occlusion problem. There is less occlusion and more space between organs in plants with spread-out structures. In our study, MBBS and FBBS both incorporate spread-out structures. Therefore, although the plants of MBBS have more branches, the data quality was similar to that of FBBS plants. For the MBCS plants, the situation was more complicated. If the branches were clustered (Fig. 13A), then there would be serious occlusion between different organs, and the quality of the laser point cloud was poor. In contrast, if the branches were spread out (Fig. 13B), then the occlusion was not serious, and we could still obtain complete data. The *Re_LM_* of c7-1 was only 60.01%, while that of c8-1 was 94.29%. More parameters need to be applied for more detailed morphological quantitative analysis, such as the dispersion of different levels of branches. Similar problems have also been found in other studies, in which occlusion makes it challenging to segment leaves and other organs [39,40], thereby affecting the inversion of the vegetation index [41,42] and the estimation of yield [43]. The occlusion effect is an emergent problem that needs to be approached for the future application of 3D phenotyping for plant populations or field scales.

### Applicability of the SHS algorithm in 3D phenotyping

The skeleton has been proven to be a piece of effective structural information for plant organ segmentation [44,45], and it has been applied to monocot plants such as sorghum and maize [44,45]. However, these methods demand the manual inclusion of prior knowledge, such as height information and initial growth points [46,47]. In addition, the plant material used in such earlier studies has been simple in structure, generally with only one main stem and no multiple branches. In contrast, oilseed rape is a dicotyledonous plant, and its structure is complex and varied. The SHS algorithm proposed in this study achieves skeleton extraction and optimization by combining the ideas of the local clustering segmentation and the growing algorithms for skeleton points and realizes the segmentation of siliques on this basis. Our method uses DBSCAN to cluster the skeleton into multiple classes and then applies the similarity of local geometric information of the same skeleton for optimization, so it can reduce the effect of other organs on its skeleton and has good performance on plants with different structures (see the Supplementary Materials). Moreover, our method requires no manual prior knowledge and is suitable for oilseed rapes of various postures. In addition, for the oilseed rapes grown in the field, there are more factors such as the interference between different plants and organs, which will be the limitation of the application of our method. It is necessary to establish the relationship model between the individual scale and the group scale to better apply our method.

In recent years, deep learning has been applied for the segmentation of plant organ point clouds with good results [48–50]. Compared with deep learning algorithms, SHS is driven by geometric rules rather than data, so it has no requirement of training a large amount of data. However, the methods based on geometric rules require researchers to have a comprehensive understanding of object morphology. For the structural diversity of oilseed rape, many special cases have been easy to ignore. In our study, the method is based on a common assumption that siliques are flat and long. Because of genetic and environmental effects, some siliques become short and wide, which will be treated as aborted siliques by our method [51]. For other plants, we need to adjust the method according to the morphological characteristics of their organs. For example, the rice tillers are slim and long, which are similar to oilseed rape branches, and our method can be applied directly for extracting tillers. As for the cotton boll, we need to design strategies to figure out the globular organs. Deep learning is driven by data, which makes it easier to understand geometric rules and extract features of morphological structures. Researchers’ tasks are to build big datasets and design robust frameworks. It is necessary to collect data on various morphological architectures and cultivars of oilseed rape plants for further research. Nevertheless, on the basis of the performance of the SHS algorithm, it can also be applied to the task of data labeling in deep learning research.

### Structural parameter relationships with oil seed yield

Seed yield is directly determined by the SN, seed number in siliques and thousand SW, and is also indirectly affected by silique traits [52–55]. For example, SL is positively correlated with seed number but poorly correlated with seed yield [52,53]. Plants differ greatly in their structures of branches and the numbers of branches, and the SN of lateral branches of a single plant has a high correlation with yield [56,57]. Moreover, many studie*s* have shown that silique structural parameters are influenced by quantitative trait loci and the environment [6,32,51,58–61]. In our study, we reached a similar conclusion that the environment had a great influence on the plant structure, and the structural differences further affected the yield of each plant. The greenhouse environment was controllable, but the cultivation and fertilization treatments were not optimal. Therefore, the greenhouse cultivars (c1 to c3) were not only small in SN and yield but also had few differences in structure. In contrast, there were many factors in the field environment, resulting in large differences in plant structure. There were 3 architectures of FBBS, MBBS, and MBCS in field cultivation. However, the reasonable control of fertilization and cultivation in the field resulted in a higher YOP. Moreover, the number of siliques, and branches and the levels of branches were different in different architectures. Among them, the plants of MBCS architecture had the most multiple branches, which resulted in more siliques and higher yield. The architecture of MBBS plants was similar to that of FBBS plants, but the former had more multiple branches and, correspondingly, more siliques and higher yields. Despite the influence of the environment, the traits of siliques (SN, SL, and SV) showed a high correlation with yield. Moreover, affected by environmental factors, such as high temperature, seed abortion can occur easily in effective siliques [62,63]. It is difficult to provide information on seed abortion inside siliques by noninvasive 3D methods at the plant scale, which affects the application of silique parameters (especially length and volume) for yield estimation. In addition, the breakage of mature siliques resulting in seed loss also affects yield estimation.

It is worth mentioning that, because of the limitations of traditional artificial or 2D image methods, the current research on oilseed rape mainly focuses on the relationship between SN, SL, and yield, while the correlation analysis of other phenotypic traits, such as SV, is limited. In this study, 3D imaging technology was used for the digitalization of plants, which could show the structural characteristics of plants more intuitively and provide for the possibility of quantitative analysis of plant structural phenotypes. In addition to SN, SL, and SV, we can further explore the phenotype information of oilseed rape to quantify traits such as the thickness of the silique canopy (see the Supplementary Materials), which are also parameters of particular interest to breeders [64]. Furthermore, 3D imaging of the total plant can retain the complete physical structure information of the plant, which provides possibilities for traceability and future exploration of potential traits.

### Conclusions

Conventional skeleton extraction requires prior knowledge or ignores the occlusion caused by complex structure limits to simple plants, which is not suitable for obtaining silique traits of oilseed rape with multiple branches. To overcome these problems, we proposed an accurate SHS method for morphological parameter extraction of oilseed rape siliques. After removing noise and non-plant data, the point cloud was skeletonized and hierarchically segmented to determine the siliques followed by morphological trait extraction. The performance of SHS has good accuracy, adaptability, and robustness for the cultivars of oilseed rape plants with 3 different structures: FBBS, MBBS, and MBCS. The average recall (*Re_EL_*) between SN*_E_* and SN*_L_* was 93.91%, 90.27%, and 83.81%, respectively. For all cultivars, the *R*^2^ of SN*_E_* and ground truth was 0.922. In addition, the traits SN, total SL, and total SV of a single plant extracted by SHS were significantly correlated with YOP, and the *R* values were 0.935, 0.916, and 0.897, respectively. Therefore, this noninvasive method could be applied for yield estimation and phenotyping analysis and provide technical support for oilseed rape breeding. Further work should focus on enriching the 3D data of oilseed rape with a larger number of different structures and combining deep learning for research.

## Data Availability

The datasets during and/or analyzed during the current study are available from the corresponding author upon reasonable request.

## Supplementary Materials

Supplementary Materials Fig. S1. Source point clouds and skeleton points (red dots) of 8 cultivars. Fig. S2. Branch length histograms of 8 cultivars. Fig. S3. The partial region data of the plants. Fig. S4. The thickness of silique canopy. Table S1. Silique segmentation results of all plants with different plant architectures.
