# Supplementary material for: Phenotyping of Silique Morphology in Oilseed Rape Using Skeletonization with Hierarchical Segmentation
Source: Plant Phenomics. 2023 Mar 15;5:0027. doi: 10.34133/plantphenomics.0027 (PMC10017417; doi:10.34133/plantphenomics.0027)
Supplement: Supplementary Materials — Fig. S1. Source point clouds and skeleton points (red dots) of 8 cultivars. Fig. S2. Branch length histograms of 8 cultivars. Fig. S3. The partial region data of the plants. Fig. S4. The thickness of silique canopy. Table S1. Silique segmentation results of all plants with different plant architectures. [file plantphenomics.0027.f1.docx]

**Supplementary Materials**

**The structures of eight cultivars**

Our study used eight cultivars to evaluate the performance of our method, including ZD619 (c1), ZD622 (c2), ZD630 (c3), Sl512 (c4), Bnw1.61/83 (c5), 8426016 (c6), CR3168 (c7) and Hu135 (c8). The skeleton points and raw data of these cultivars were shown in Fig. 4

| 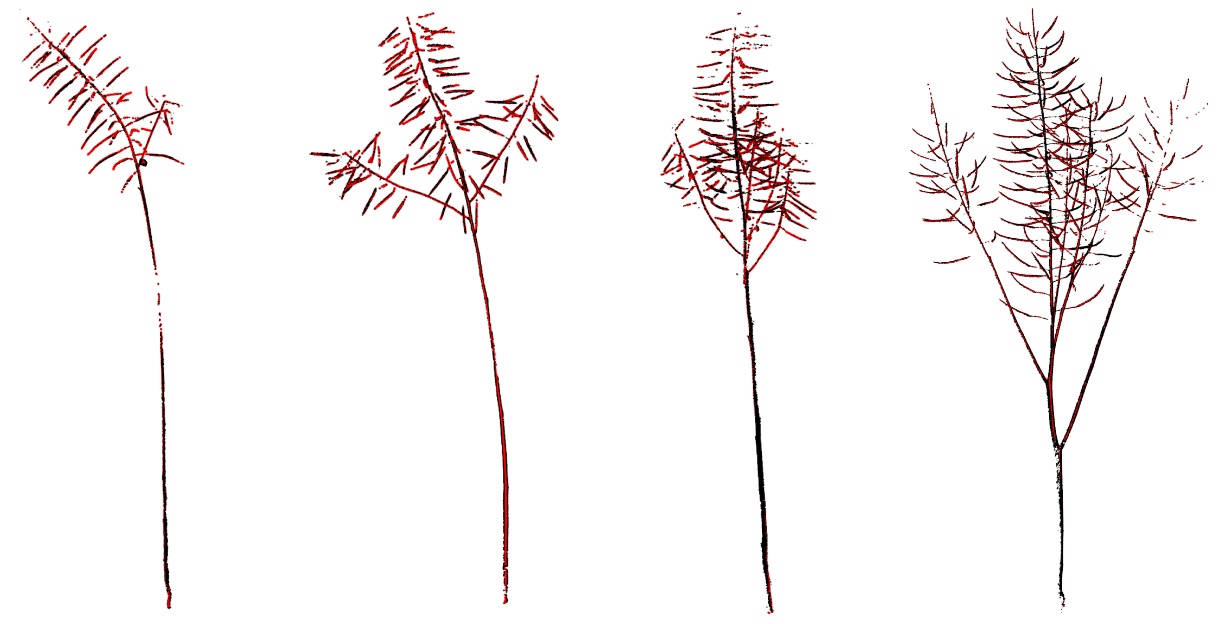 | | | |
| --- | --- | --- | --- |
| ZD619(c1) | ZD622(c2) | ZD630(c3) | Sl512 (c4) |
| 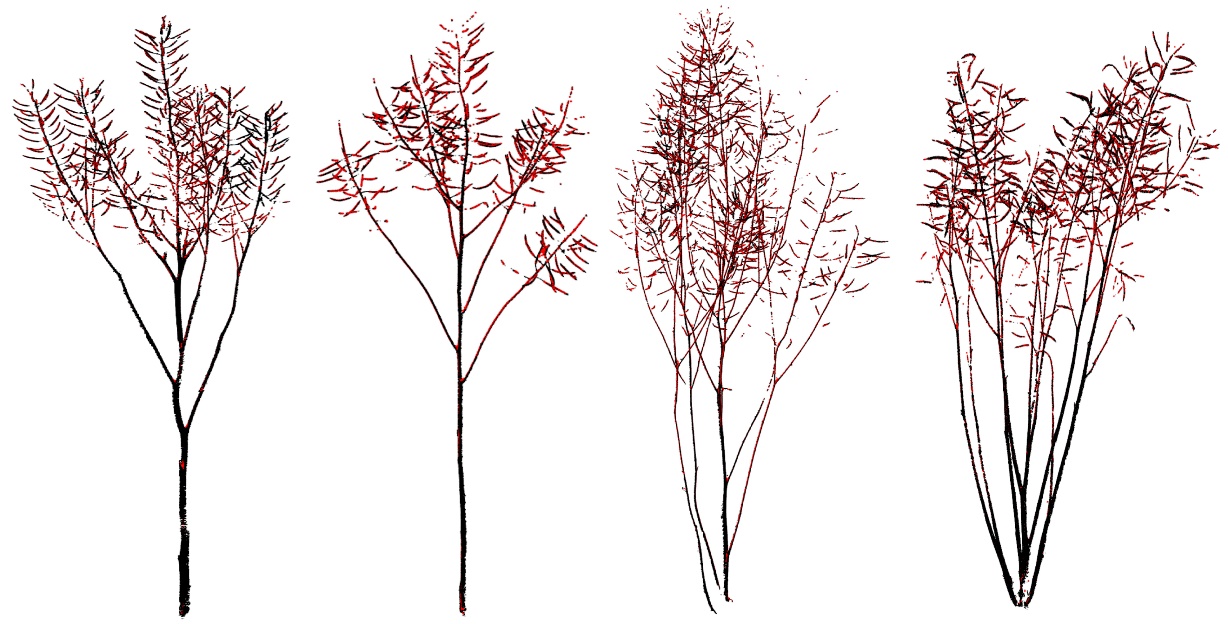 | | | |
| Bnw1.61/83 (c5) | 8426016 (c6) | CR3168 (c7) | Hu135(c8) |

Fig. 1 Source point clouds and skeleton points (red dots) of eight cultivars

**The siliques segmentation results of eight cultivars**

The histograms of the sub-skeleton length of three plant architectures were shown in Fig. 5. For all cultivars, the length distribution of effective siliques of a plant obeyed normal distribution (Fig. 5c).


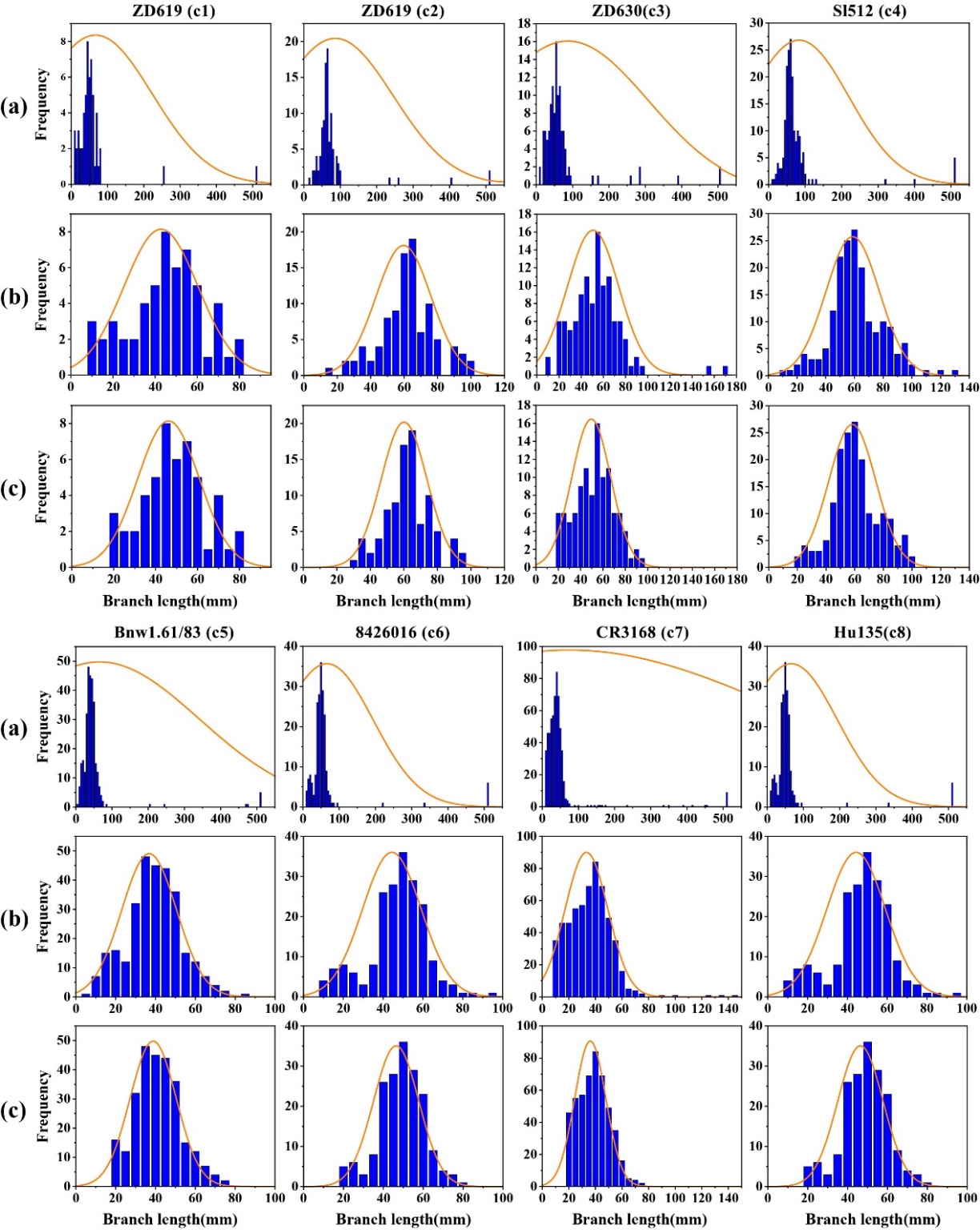


Fig. 2 Branch length histograms of eight cultivars. (a) The length distribution of all branches including stems and siliques, and the branches longer than 500mm are combined into one group for display convenience. (b) The length distribution of branches without long stems. (c) The length distribution of valid siliques.

The plant architectures and silique segmentation results of all samples of these eight cultivars were shown in Table 1 and Fig. 6. Among them, c4 and c6 both had two types of architecture: FTBS and MTBS. Cultivar c8 also had two types: MBBS and MBCS. And the segmented siliques were rendered with unique colors.

**Table 1 Silique segmentation results of all plants with different plant architectures**

| Plant architecture | Samples | ${NB}_{1st}$ | ${NB}_{2nd}$ | ${NB}_{3rd}$ | ${SN}_{M}$ | ${SN}_{L}$ | ${SN}_{E}$ | ${Re}_{LM}$ (%) | ${Re}_{EL}$ (%) | ${Re}_{EM}$ (%) |
| --- | --- | --- | --- | --- | --- | --- | --- | --- | --- | --- |
| FBBS | c1-1 | 1 | 3 | 0 | 53 | 53 | 50 | 100.00 | 94.34 | 94.34 |
|  | c1-3 | 1 | 2 | 0 | 41 | 41 | 40 | 100.00 | 97.56 | 97.56 |
|  | c1-4 | 1 | 1 | 0 | 42 | 42 | 41 | 100.00 | 92.86 | 97.62 |
|  | c2-1 | 1 | 1 | 0 | 41 | 41 | 37 | 100.00 | 92.68 | 90.24 |
|  | c2-2 | 1 | 4 | 0 | 129 | 103 | 91 | 79.84 | 93.20 | 70.54 |
|  | c2-3 | 1 | 3 | 0 | 74 | 71 | 70 | 95.95 | 98.59 | 94.59 |
|  | c3-1 | 1 | 4 | 0 | 104 | 100 | 89 | 96.15 | 91.00 | 85.57 |
|  | c3-2 | 1 | 6 | 0 | 129 | 125 | 108 | 96.90 | 81.60 | 83.72 |
|  | c3-3 | 1 | 4 | 0 | 78 | 71 | 65 | 91.03 | 88.73 | 83.33 |
|  | c4-1 | 1 | 4 | 0 | 175 | 172 | 170 | 98.29 | 98.84 | 97.14 |
|  | c4-2 | 1 | 4 | 1 | 214 | 184 | 183 | 85.98 | 99.46 | 85.51 |
|  | c6-1 | 1 | 6 | 0 | 196 | 185 | 183 | 94.39 | 98.92 | 93.37 |
|  | c6-2 | 1 | 6 | 0 | 226 | 217 | 202 | 96.02 | 93.09 | 89.38 |
|  | Average | 1 | 3.69 | 0.08 | 115.54 | 108.08 | 102.23 | 94.97 | 93.91 | 82.50 |
|  | SD | 0 | 1.64 | 0.27 | 65.61 | 60.23 | 58.81 | 5.85 | 4.85 | 7.42 |
| MBBS | c4-3 | 2 | 7 | 7 | 388 | 371 | 352 | 95.62 | 94.88 | 90.72 |
|  | c5-1 | 1 | 6 | 3 | 295 | 286 | 258 | 96.95 | 90.21 | 87.46 |
|  | c5-2 | 1 | 6 | 3 | 406 | 373 | 310 | 91.87 | 83.11 | 76.35 |
|  | c5-3 | 1 | 7 | 8 | 439 | 388 | 348 | 88.38 | 89.69 | 79.27 |
|  | c6-3 | 1 | 7 | 3 | 270 | 255 | 229 | 94.44 | 89.80 | 84.81 |
|  | c8-3 | 2 | 5 | 2 | 99 | 99 | 93 | 100.00 | 93.94 | 93.94 |
|  | Average | 1.33 | 6.33 | 4.33 | 316.17 | 295.33 | 265.00 | 93.45 | 90.27 | 85.43 |
|  | SD | 0.47 | 0.75 | 2.29 | 114.06 | 100.50 | 88.89 | 3.69 | 3.80 | 6.13 |
| MBCS | c7-1 | 4 | 15 | 4 | 577 | 352 | 292 | 61.01 | 82.95 | 50.61 |
|  | c7-2 | 3 | 11 | 10 | 615 | 484 | 386 | 78.70 | 79.75 | 62.76 |
|  | c7-3 | 3 | 12 | 7 | 673 | 573 | 473 | 85.14 | 82.55 | 70.28 |
|  | c8-1 | 6 | 21 | 5 | 455 | 429 | 400 | 94.29 | 93.24 | 87.91 |
|  | c8-2 | 5 | 12 | 5 | 428 | 396 | 319 | 92.52 | 80.56 | 74.53 |
|  | Average | 4.20 | 14.2 | 6.2 | 549.6 | 446.8 | 374.00 | 82.33 | 83.81 | 69.22 |
|  | SD | 1.17 | 3.66 | 2.14 | 93.80 | 76.41 | 63.83 | 12.02 | 4.86 | 12.38 |
| Average | / | / | / | / | / | / | / | 92.23 | 90.90 | 84.23 |
| SD | / | / | / | / | / | / | / | 8.82 | 6.07 | 11.54 |
| Note: ${SN}_{M}$ was measured silique number by manual; ${SN}_{L}$ was the silique number counted by laser point cloud; ${SN}_{E}$ was silique number estimated by our method; SD was standard deviation; ${Re}_{LM}$ was recall = ${SN}_{L}/{SN}_{M}$, ${Re}_{EL}$ was recall = ${SN}_{E}/{SN}_{L}$,${Re}_{EM}$ was recall = ${SN}_{E}/{SN}_{M}$. $NB_{1st}$ was the number of the first branch.${NB}_{2nd}$ was the number of the second branch. ${NB}_{3rd}$ was the number of the third branch. | | | | | | | | | | |

| 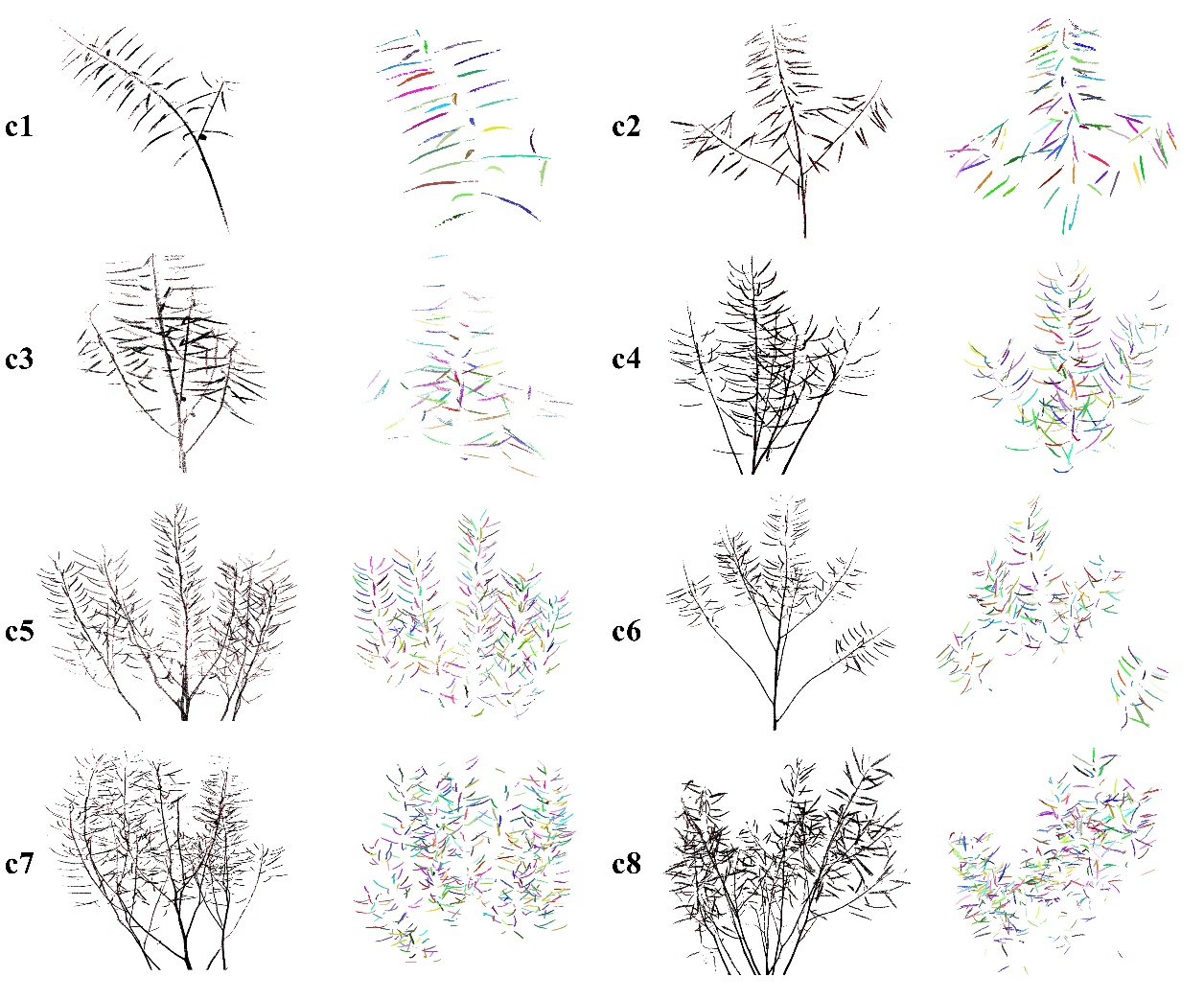 | | | |
| --- | --- | --- | --- |
| (a) | (b) | (a) | (b) |

Fig. 3 The partial region data of the plants. (a) The skeleton connecting lines combined with point clouds of tillers and siliques. (d) The siliques point cloud, and each silique has its own unduplicated individual color.

**The parameters of oilseed rape**

Based on the results of silique segmentation, we could also obtained some important parameters, like the thickness of silique canopy (Fig. 4)


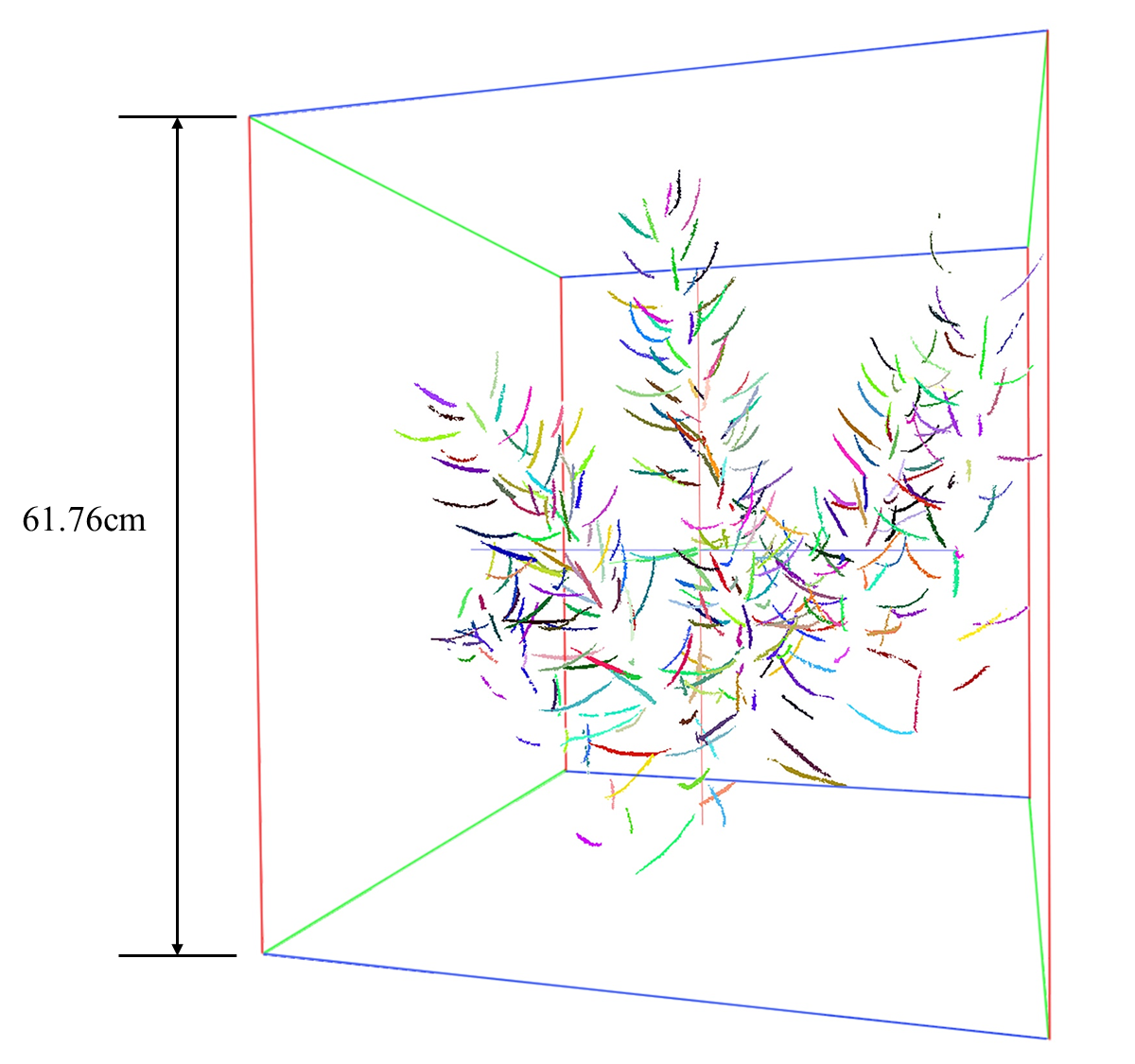


Fig. 4 The thickness of silique canopy
